# Chemoselective bond activation by unidirectional and asynchronous PCET using ketone photoredox catalysts†

**DOI:** 10.1039/d3sc04362b

**Published:** 2023-11-02

**Authors:** Rui Sun, Serge Ruccolo, Daniel L. Nascimento, Yangzhong Qin, Nathaniel Hibbert, Daniel G. Nocera

**Affiliations:** a Department of Chemistry and Chemical Biology, Harvard University 12 Oxford Street Cambridge MA 02138 USA dnocera@fas.harvard.edu

## Abstract

The triplet excited states of ketones are found to effect selective H-atom abstraction from strong amide N–H bonds in the presence of weaker C–H bonds through a proton-coupled electron transfer (PCET) pathway. This chemoselectivity, which results from differences in ionization energies (IEs) between functional groups rather than bond dissociation energies (BDEs) arises from the asynchronicity between electron and proton transfer in the PCET process. We show how this strategy may be leveraged to achieve the intramolecular anti-Markovnikov hydroamidation of alkenes to form lactams using camphorquinone as an inexpensive and sustainable photocatalyst.

## Abstract 2

The triplet excited states of ketones effect selective H-atom abstraction from amide N–H bonds in the presence of weaker C–H bonds *via* a proton-coupled electron transfer (PCET) pathway in which the electron and proton transfers are asynchronous.

## Graphical abstract

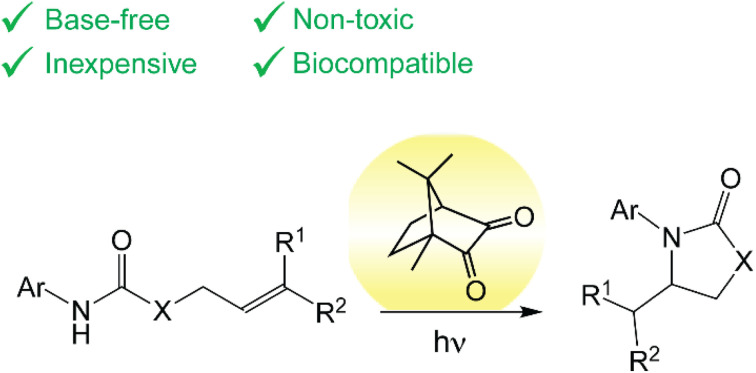

## Introduction

**Fig. 1:**
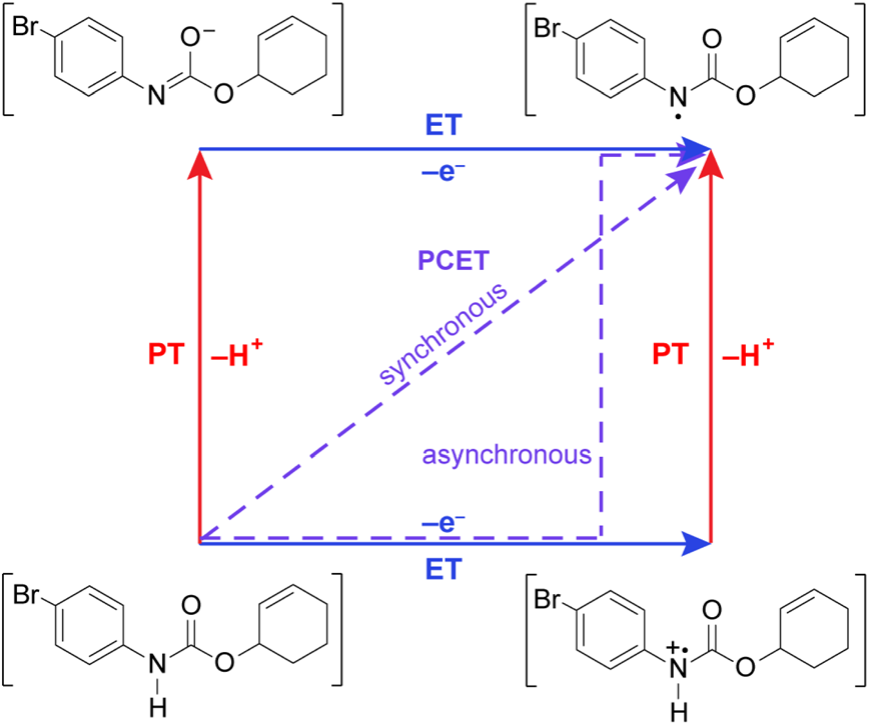
Square scheme highlighting synchronous and asynchronous PCET pathways for substrate activation.

Leveraging proton-coupled electron transfer (PCET) as a foundational design element of photoredox methods has led to a powerful strategy for the selective generation of highly reactive organic intermediates such as heteroatom-centered radicals (*X*˙). As illustrated in Fig. 1 for the generation of a nitrogen radical from an amide, the PCET event may be described in terms of four diabatic states as accommodated by a “square scheme”.^1,2^ A discrete intermediate is formed at the corners of the square scheme due to stepwise electron transfer followed by proton transfer (ET-PT) or *vice versa* (PT-ET). The ET-PT and PT-ET paths along the edges are characterized by two transition states: one for proton transfer and one for electron transfer. Anywhere within the square scheme, PCET is characterized by a single transition state, whether the PCET pathway is synchronous (along the diagonal, *e.g.*, hydrogen atom transfer) or asynchronous (*i.e.*, zig-zag). Asynchronous PCET is common to bidirectional PCET, wherein the proton and electron are transferred to different acceptors. Conversely, asynchronous or synchronous PCET may occur for unidirectional PCET, wherein the proton and electron are transferred to the same acceptor.^2–5^

**Fig. 2:**
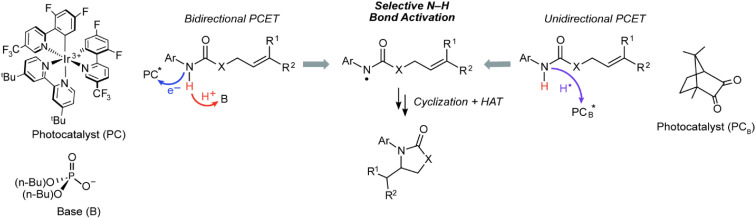
Photoredox intramolecular hydroamidation reaction promoted by bidirectional and unidirectional PCET. The bidirectional PCET occurs by an outer-sphere electron transfer to a photoexcited acceptor (PC*) followed by proton transfer to an exogenous base (left pathway). Typical examples of photocatalyst and base used in bidirectional PCET are shown. Unidirectional PCET occurs when the photoredox reagent, PC_B_*, is the electron and proton acceptor, such as the triplet excited state of ketones (right pathway, this work).

Bidirectional and unidirectional PCET mechanisms have been utilized for substrate activation. Bidirectional PCET has been particularly useful for the design of chemoselective photoredox methods to generate *X*˙ in organic molecules containing C–H bonds whose bond dissociation energies (BDEs) are much lower than those of the corresponding X–H bonds.^6–13^ For such methods (Fig. 2, left pathway), an electron is transferred to a photocatalyst (PC*), such as an Ir/Ru polypyridyl or cyclometallated complex,^14–16^ and the proton is accepted by either an exogenous base or basic functionality on the ligand. The bidirectional PCET pathway has been especially fruitful for the selective photogeneration of amidyl radicals (N–H BDE of ∼100 kcal mol^−1^),^6^ which may add to olefins (allylic C–H BDE of ∼83 kcal mol^−1^)^7^ to furnish anti-Markovnikov products in exceptional yields.^6,17,18^ Alternatively, for unidirectional PCET (Fig. 2, right pathway), the proton and electron are both transferred to the photocatalyst, PC_B_*. Unidirectional PCET offers the advantage of decreased molecularity and inherently higher reaction rates compared to bidirectional PCET, leading, in principle, to a higher energy efficiency. Examples of the application of unidirectional PCET include the photogeneration of halogen radicals from earth abundant metal complexes,^19–22^ which have been identified as key intermediates in the PCET activation of C(sp^3^)–H bonds for alkylation,^23–26^ alkenylation,^27^ arylation,^19,28,29^ acylation,^19,30^ and amination^31,32^ reactions. Notwithstanding, the activation of substrates by these compounds is predominantly dictated by thermodynamic bond strengths modulated by steric and polarity effects, leading to inferior control of chemoselectivity as compared to that achieved in bidirectional PCET systems.

Photoexcited states of ketones are known to undergo unidirectional PCET *via* their conspicuous hydrogen atom transfer photochemistry, leading to their ubiquitous application as photoinitiators in polymerizations^33–42^ and, more recently, as catalysts for photoredox reactions.^43,44^ However, in contrast to the striking chemoselective activation of strong X–H bonds afforded by bidirectional PCET, much of the reactivity derived from ketone photoreagents has been limited to abstraction of weak C–H bonds adjacent to aryl or heteroatomic functionality.^45^ We now report that the photoexcited states of certain ketones, such as camphorquinone (CQ), are capable of selectively abstracting a strong amide X–H bond in the presence of much weaker C–H bonds, thus enabling the chemoselective generation of amidyl radicals (Fig. 2, right). Mechanistic studies establish that such chemoselectivity is the result of an asynchronous unidirectional PCET process where the quenching of a CQ excited state (CQ*) primarily correlates with the ionization energy (IE) of the substrate as opposed to its BDE. Additionally, the approach of utilizing ketone organo-photocatalysts has the added benefit of much lower toxicity in comparison to common photocatalysts based on noble metals such as Ir, the concentrations of which are strictly regulated in drug products^46–50^ (*e.g.*, 0.5 ppm for parenteral administration and 5 ppm for oral administration in the case of Ir).

## Results and discussion

**Fig. 3:**
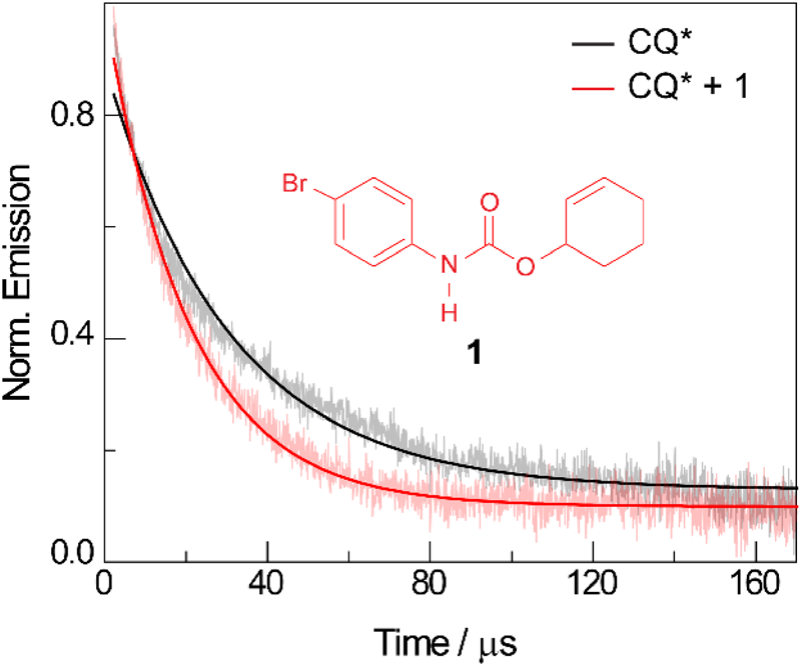
Time-resolved emission decay traces monitored at 570 nm for a DCM solution of CQ (5 mM) in the absence (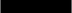 black) and presence (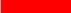 red) of amide 1 (0.4 mM). The excited state lifetimes extracted from monoexponential fits of the data were 30.6 μs and 20.7 μs, respectively. *λ*_exc_ = 460 nm.

**Fig. 4:**
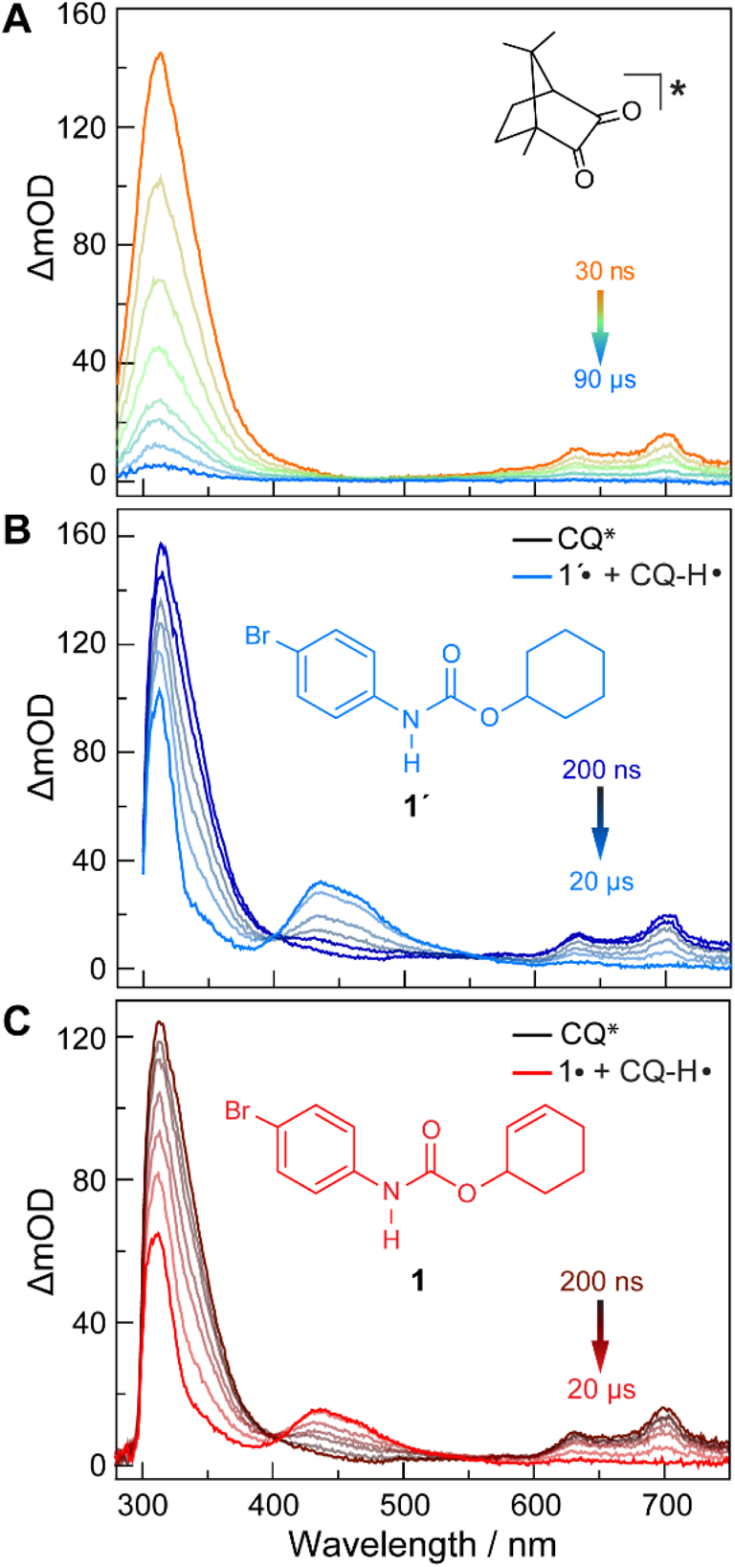
TA spectra of DCM solutions containing CQ (5 mM) and amide substrates (10 mM) in DCM: (A) for a solution of CQ alone. (B) For a solution of CQ with amide 1′ as the substrate. (C) For a solution of CQ with amide 1 as the substrate. *λ*_exc_ = 460 nm.

The quenching of CQ* by amide substrate is responsible for amidyl radical formation. Known amide substrates were either purchased or prepared as previously described,^6^ whereas new substrates were synthesized and characterized (Fig. S1–S7†) as reported in Section B of the ESI.† As shown in Fig. 3, the lifetime of CQ* (as measured by time-resolved emission kinetics at 570 nm) decreases from 30.6 μs to 20.7 μs upon addition of amide 1 (0.4 mM), implying quenching of the former by the latter. Transient absorption (TA) spectroscopy permits this reaction between amide 1 and CQ* to be directly interrogated. We focused on this initial quenching step because the subsequent steps leading to cycloamidation (*i.e.*, cyclization and subsequent HAT to furnish the lactam) occur independently of the photocatalyst.^51^Fig. 4 shows the transient absorption spectra for solutions containing CQ (5 mM) alone and those containing CQ with amides 1 and 1′ (10 mM). The spectra of CQ in Fig. 4A show the relaxation of CQ*,^52^ while the spectra for CQ in the presence of 1′ and 1 in Fig. 4B and C, respectively, show initial absorbance dominated by the excited state of CQ at 200 ns (black traces), followed by a gradual evolution to a spectrum containing features at 430 nm and 310 nm (blue trace for 1′ and red trace for 1). The peak at 430 nm is ascribed to the amidyl radical^51^ while the 310 nm feature is tentatively assigned to CQ–H^•^ due to its resemblance to the spectrum of CQ^•–^ obtained by spectroelectrochemistry (Fig. S8B†) as well as a previously reported transient feature observed during the photoreduction of CQ by isopropanol.^53^ Fig. S10† shows the TA kinetic trace at 430 nm for samples containing CQ and amide substrate 1′, which is identical to substrate 1 with the exception of an olefin moiety. Substrate 1′ is strategic because it is unable to undergo cyclization upon amidyl radical formation, thus allowing for the kinetics of forward and back HAT reactions to be measured without interference from other chemical processes. From kinetic modelling of the decay of the transient absorption at 430 nm (Fig. S10†), we extract an HAT rate constant of *k*_FH˙_ = 2.9 × 10^7^ M^−1^ s^−1^ and a back reaction rate constant of *k*_BH˙_ = 8.3 × 10^9^ M^−1^ s^−1^, where the latter is similar to the back-electron transfer rate constant measured for the Ir/base-catalyzed system (*k*_BET_ = 7.9 × 10^9^ M^−1^ s^−1^).^51^ We note that there is negligible ground-state hydrogen bonding between CQ and amide 1, as the association constant between the two was determined to be *K*_a_ = 2.4 ± 0.2 M^−1^ by ^1^H NMR spectroscopy (Fig. S11†). This implies that the chemoselectivity for amidyl radical formation is intrinsic to the excited state reactivity of CQ*.

In order to differentiate between stepwise and concerted mechanisms in the generation of amidyl radical by CQ*, we investigated the relative quenching of CQ* by a series of Ph–XH (*X* = NH, S, and O) compounds and their X-methylated derivatives. Since all the Ph–XH substrates show irreversible oxidation waves, we used the gas-phase ionization energies (IEs) of these compounds as a measure of their oxidation potential, as has been previously discussed for asynchronous PCET pathways.^54^ Table S1† lists the calculated quenching rate constants (*k*_q_) for these compounds determined from Stern–Volmer plots (Fig. S12†) along with their IEs, X–H BDEs, and p*K*_a_ values in DMSO. If a PT-ET mechanism were operative, a correlation between *k*_q_ and p*K*_a_ is expected since the quenching would be governed by proton transfer. However, this is not the case. We observe that the *k*_q_ values correlate with IEs, signifying the importance of ET character in the quenching process. To confirm that the quenching of CQ* by Ph–XH substrates leads to X–H bond homolysis, we employed TA spectroscopy to study the reaction between CQ (10 mM) and phenol (20 mM) in DCM. Under these conditions, we observed the clear formation of phenoxyl radical with features at 380 and 400 nm (Fig. S9†).^55^

**Fig. 5:**
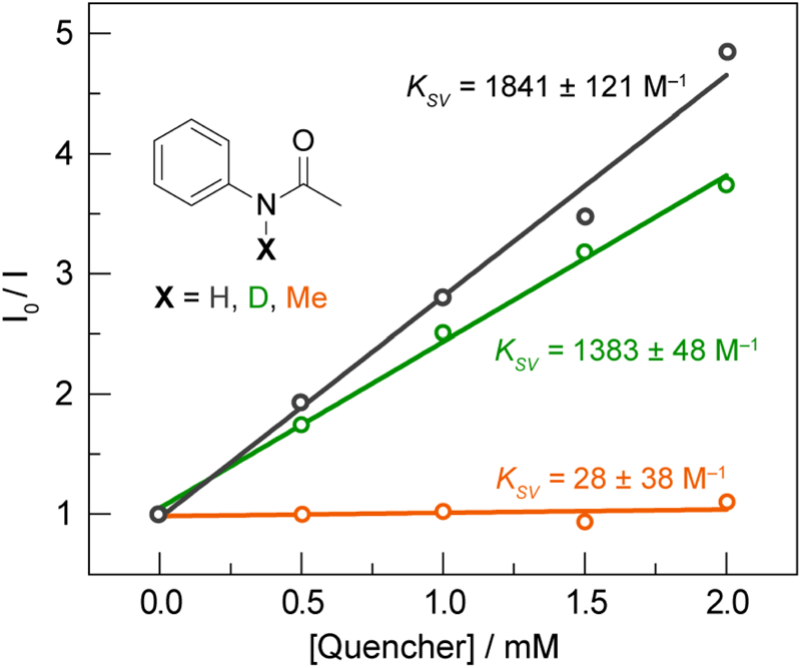
Stern–Volmer plot for the quenching of CQ (1 mM) by acetanilide (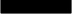 black), deutero-acetanilide (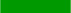 green) and *N*-methylacetanilide (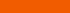 orange) in DCM.

To further delineate between the stepwise ET-PT and concerted asynchronous PCET pathways, we first note that CQ* has an oxidation potential of 0.33 V *vs.* Fc^+^/Fc, based on a E(CQ/CQ•^−^) = −1.90 V *vs.* Fc^+^/Fc (Fig. S8A†) and the previously reported excited state energy of 2.23 eV for CQ* at 77 K.^56^ As CQ* is a far weaker outer-sphere photooxidant than the Ir catalyst (oxidation potential of 0.85 V),^51^ which is not quenched by the amide substrate in the absence of base,^6^ a stepwise ET-PT pathway for amidyl radical formation is unfeasible based on the redox potential of CQ*. This is further corroborated by a comparison of the Stern–Volmer constants (*K*_*SV*_) for acetanilide and *N*-methylacetanilide. The latter is expected to have a similar or lower oxidation potential for outer-sphere ET when compared to the former; however, only the former possesses a proton that can engage in a PCET process. As shown in Fig. 5, the Stern–Volmer constant for *N*-methylacetanilide (*K*_*SV*_ = 28(38) M^−1^, *k*_q_ = 9 (12) × 10^5^ M^−1^ s^−1^) is two orders of magnitude lower than for acetanilide [*K*_SV_ = 1841(121) M^−1^, *k*_q_ = 6.0 (0.4) × 10^7^ M^−1^ s^−1^], which suggests that the quenching is not dictated purely by an ET process followed by PT. This is further supported by a KIE of *k*_H_*/k*_D_ = 1.33 (0.13) between acetanilide and acetanilide-*d*, suggesting proton involvement in the quenching process. We note that a similar KIE exists for the quenching rates of CQ* by phenol [*k*_q_ = 3.18 (0.14) × 10^9^ M^−1^ s^−1^] and phenol-d_6_ [*k*_q_ = 2.07 (0.13) × 10^9^ M^−1^ s^−1^] in DCM, wherein we measured a KIE of *k*_H_*/k*_D_ = 1.54 (0.06). Additionally, we found that 1,2,4,5-tetramethylbenzene (1,2,4,5-TMB) does not quench CQ* though its IE (8.1 eV)^57^ is approximately in line with the Ph–XH compounds in Table S1.† For a stepwise process wherein ET is followed by PT, we would expect activation of 1,2,4,5-TMB. However, in contrast to 1,2,4,5-TMB, ET originates from a site carrying proton for the Ph–XH substrates listed in Table S1.† For these substrates, the p*K*_a_ of the proton is expected to decrease substantially with oxidation and hence consistent with p*K*_a_ contributing to the kinetics of the overall quenching.

Taken together, the quenching and kinetic isotope effects are most consistent with a concerted asynchronous PCET pathway with a transition state that is predominantly ET in character, but does not involve the generation of distinct, oxidized intermediate preceding proton transfer (as shown in the asynchronous pathway delineated in Fig. 1). This mechanism explains the chemoselectivity for amide N–H bond activation over allylic C–H bonds, since the IEs for the former are much lower than those for the latter (*e.g.*, 8.2 eV for 4′-fluoroacetanilide *vs.* 8.9–9.1 eV for cyclohexene),^57^ in addition to being less acidic. Furthermore, we note that a concerted asynchronous PCET between amide and carbon nitride may also account for the background hydroamidation reactivity observed with carbon nitride photocatalysts in the absence of base.^58^

To demonstrate the synthetic utility of the pathway shown in Fig. 2 (right), we sought to establish whether CQ itself can serve as a competent photocatalyst in intramolecular hydroamidation reactions in the absence of an exogenous base. As shown in Entry 1 of Table 1, cyclized product 3 can be formed from 1 in 94% yield after 24 h of blue LED irradiation using 20% CQ and 10% phenyl disulfide (PhSSPh) as a hydrogen atom shuttle to facilitate turnover of CQ–H^•^ and intercept, through the intermediacy of thiophenol, the transient carbon-centered radical formed after cyclization of the amidyl radical. We measured a quantum yield of *Φ* = 0.1 for this reaction, which is on par with that of the Ir-catalysed reaction. The omission of disulfide (entry 2) or its replacement with thiol (entry 3) led to significantly diminished yields, consistent with previous observations under Ir-catalyzed conditions.^51,59^ Attenuated yield was also observed for the methoxy-substituted substrate 2 (Entry 4), which has been shown to undergo cyclization at a rate that is three orders of magnitude slower upon amidyl radical formation when compared to 1.^51^ However, by switching from PhSSPh to 2,4,6-triisopropylphenyl disulfide [(TripS)_2_], we observed significantly improved yields for substrate 2 (entry 5). This superior performance of [(TripS)_2_] as compared to its phenyl congener has previously been documented in photoredox reactions,^18,60^ and is possibly due to steric protection afforded by the isopropyl moieties leading to a higher steady-state concentration of thiyl radical *via* retardation of disulfide formation. Finally, we investigated the performance of CQ-mediated hydroamidation in acetonitrile (MeCN), a highly polar solvent. The original method relying on bidirectional PCET using an outer-sphere Ir photooxidant and a phosphate base necessitated the coalescence of Ir*, base, and amide substrate in order to generate the amidyl radical. This is aided by ion pairing between the cationic Ir photooxidant and anionic phosphate base in DCM,^51^ which can be disrupted by a highly polar solvent. Since CQ is a neutral species which does not rely on ion pairing effects for its unidirectional PCET activity, we posited that it could deliver superior yields in MeCN. Indeed, as shown in entries 6 and 7, the use of CQ results in a yield that was ∼3× higher than that with the Ir/base system; switching from PhSSPh to (TripS)_2_ further resulted in a substantial increase in the yield to 43% (entry 8).

**Table 1:** Optimization of the CQ-mediated intramolecular cycloamidation of alkenes

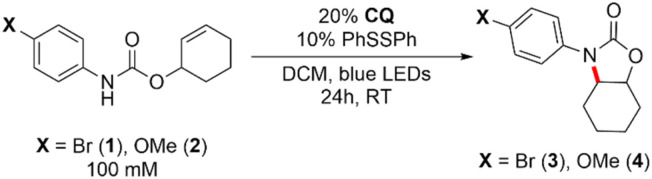			
Entry	X Group	Differences from standard conditions listed above	Yield^a^ (%)
1	Br	None	94
2	Br	No PhSSPh	9
3	Br	PhSH instead of PhSSPh	45
4	OMe	None	32
5	OMe	(TripS)_2_ in place of PhSSPh	51
6	Br	MeCN in place of DCM	14
7	Br	Ir photooxidant + phosphate base^b^, MeCN in place of DCM	<5
8	Br	(TripS)_2_ in place of PhSSPh, MeCN in place of DCM	43

a Yield determined by ^1^H NMR spectroscopy.

b Same conditions as the published procedure, ref. 6, with 10% PhSSPh in place of 20% PhSH for consistency with CQ-mediated conditions. The phosphate base is [NMeBu_3_][OP(O)(*n-*BuO)_2_]. Trip = 2,4,6-triisopropylphenyl.

To investigate the stability of CQ in the reaction and verify its role as the active photocatalyst,^51^ we measured the yield of cyclized product 4 and compared with the amount of CQ remaining by ^1^H NMR spectroscopy. Fig. S13† (red traces, (TripS)_2_ as disulfide) shows that no increase in product yield was observed after CQ was completely consumed. Analysis of the reaction mixture after photolysis by mass spectrometry (ESI-MS) revealed the formation of hydroxycamphor and camphanediol as two possible CQ decomposition products. However, *ca.* 20% product formation did occur when *ca.* 95% CQ was consumed in the presence of (TripS)_2_. This could be due to the presence of intermediate photoproducts (*e.g.*, thioamide species)^51^ which can undergo further photolysis to yield the lactam. With PhSSPh as the disulfide, a much slower reaction was observed (Fig. S13,† black traces), consistent with the lower yield shown in Table 1.

Given the ubiquity of ketones as HAT photoinitiators, we sought to establish whether the selective generation of amidyl radicals *via* activation of the amide N–H bond in the presence of weak C–H bonds might be a general phenomenon. To this end, we used the cycloamidation reaction as an assay for amidyl radical generation. Although CQ remained the highest yielding ketone among those examined, a wide range of mono- and diketones gave significant yields of the cyclized product 3 (Fig. S14†), as determined by ^1^H NMR spectroscopy. Surprisingly, several commonly employed photoinitiators, which have been extensively studied for their propensity to readily undergo C–H abstraction, such as diacetyl^61–63^ and acetophenone^64–66^ gave significant yields of product, with the balance of the reaction being accounted for by unreacted starting material. These results demonstrate that chemoselectivity in hydroamidation photoredox transformations promoted by the PCET chemistry of triplet ketones is not limited to CQ.

To confirm the generality of the CQ-catalyzed hydroamidation reaction, we tested multiple substrates under the optimized conditions in Table 1. As shown in Table 2, a variety of alkene-bearing amides undergo hydroamidation under CQ photocatalysis. For more challenging substrates, (TripS)_2_ may be used in place of PhSSPh to improve the yield. Of note, Lewis acidic functionality, such as the pinacolboranyl (Bpin) moiety, was well-tolerated. Finally, CQ achieved twice the yield of the Ir + base combination in the reaction of an anionic substrate containing a trifluoroborate functional group, further highlighting the distinct reactivity of a unidirectional PCET catalyst under conditions where ion pairing between the cationic Ir photooxidant and anionic phosphate base can be disrupted. These results are of synthetic relevance as the Bpin and trifluoroborate functional groups are commonly found in nucleophilic substrates for Suzuki–Miyaura cross-coupling reactions.^67–69^ Currently, *N*-alkyl derivatives are not amenable towards cyclization under these conditions as no quenching of CQ* was observed *via* Stern–Volmer experiments with *N*-alkyl amides.

**Table 2:** Scope of the CQ-mediated intramolecular alkene hydroamidation reaction

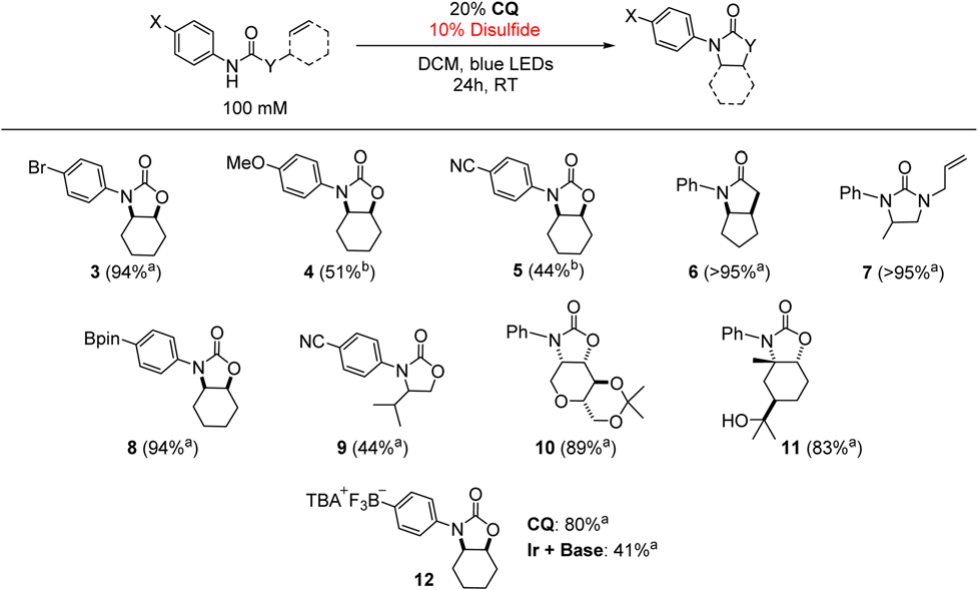

a PhSSPh used as the disulfide.

b (TripS)_2_ used as the disulfide. Yields determined by ^1^H NMR spectroscopy using 1,4-bis(trifluoromethyl)benzene or 1,3,5-tris(trifluoromethyl)benzene as an internal standard. [NMeBu_3_][OP(O)(*n*-BuO)_2_]used as base. Structures of the dominant diastereomers (as determined by crude ^1^H NMR spectroscopy with reference to previously reported spectra^6^) are drawn where appropriate.

## Conclusions

The excited states of ketones exhibit an inherent selectivity for amide N–H activation over weaker C–H bonds, as confirmed by Stern–Volmer and transient absorption experiments. This selectivity results from an asynchronous PCET reaction mechanism where the reactivity is largely dictated by the ionization energy of the functional group. This mechanism may be exploited to catalyze the intramolecular hydroamidation of alkenes under photoredox conditions with ketones including camphorquinone, which has the added benefit of being an inexpensive and non-toxic diketone,^70^ leading to a greener reaction method.

## Data availability

The ESI includes experimental for the preparation of new substrates and their ^1^H NMR spectra, 2D NMR spectra supporting assignment of relative stereochemistry for certain substrates, electrochemistry of CQ, transient absorption spectra and kinetics traces, ^1^H NMR spectra for investigating ground state H-bonding between CQ and 1, Stern–Volmer plots, photochemical results using various ketones, and details for quantum yield measurement.†

## Author contributions

RS, SR, DLN, and NH performed synthesis, reactivity studies, and characterization. RS and SR performed Stern–Volmer and (spectro)electrochemical measurements. YQ performed time-resolved laser experiments. RS, SG and DGN designed experiments. RS and DGN wrote the manuscript. DGN acquired funding for the studies reported herein.

## Conflicts of interest

There are no conflicts to declare.

## Supplementary Material

SC-014-D3SC04362B-s001

## References

[cit1] Nocera D. G. (2022). J. Am. Chem. Soc..

[cit2] Cukier R. I., Nocera D. G. (1998). Annu. Rev. Phys. Chem..

[cit3] Reece S. Y., Nocera D. G. (2009). Annu. Rev. Biochem..

[cit4] Chang C. J. , Brown J. D. K., Chang M. C. Y., Baker E. A. and Nocera D. G., Electron Transfer in Chemistry, ed. V. Balzani, Wiley-VCH, Weinheim, Germany, 2001, vol. 3, ch. 2.4, pp. 409–461

[cit5] Darcy J. W., Koronkiewicz B., Parada G. A., Mayer J. M. (2018). Acc. Chem. Res..

[cit6] Miller D. C., Choi G. J., Orbe H. S., Knowles R. R. (2015). J. Am. Chem. Soc..

[cit7] Khursan S. L., Mikhailov D. A., Yanborisov V. M., Borisov D. I. (1997). React. Kinet. Catal. Lett..

[cit8] Murray P. R. D., Cox J. H., Chiappini N. D., Roos C. B., McLoughlin E. A., Hejna B. G., Nguyen S. T., Ripberger H. H., Ganley J. M., Tsui E., Shin N. Y., Koronkiewicz B., Qiu G., Knowles R. R. (2021). Chem. Rev..

[cit9] Zhu Q., Graff D. E., Knowles R. R. (2018). J. Am. Chem. Soc..

[cit10] Choi J. G., Zhu Q., Miller D. C., Gu C. J., Knowles R. R. (2016). Nature.

[cit11] Ota E., Wang H., Frye N. L., Knowles R. R. (2019). J. Am. Chem. Soc..

[cit12] Weinberg D. R., Gagliardi C. J., Hull J. F., Murphy C. F., Kent C. A., Westlake B. C., Paul A., Ess D. H., McCafferty D. G., Meyer T. J. (2012). Chem. Rev..

[cit13] Hoffmann N. (2017). Eur. J. Org Chem..

[cit14] Wenger O. S. (2013). Acc. Chem. Res..

[cit15] Wenger O. S. (2015). Coord. Chem. Rev..

[cit16] Sinha N., Yaltseva P., Wenger O. S. (2023). Angew. Chem., Int. Ed..

[cit17] Margrey K. A., Nicewicz D. A. A. (2016). Acc. Chem. Res..

[cit18] Nguyen S. T., Zhu Q., Knowles R. R. (2019). ACS Catal..

[cit19] Shields B. J., Doyle A. G. (2016). J. Am. Chem. Soc..

[cit20] Troian-Gautier L., Turlington M. D., Wehlin S. A. M., Maurer A. B., Brady M. D., Swords W. B., Meyer G. J. (2018). Chem. Rev..

[cit21] Gygi D., Gonzalez M. I., Hwang S. J., Xia K. T., Qin Y., Johnson E., Gygi F., Chen Y.-S., Nocera D. G. (2021). J. Am. Chem. Soc..

[cit22] Gonzalez M. I., Gygi D., Qin Y., Zhu Q., Johnson E. J., Chen Y.-S., Nocera D. G. (2022). J. Am. Chem. Soc..

[cit23] Treacy S. M., Rovis T. (2021). J. Am. Chem. Soc..

[cit24] Kang Y. C., Treacy S. M., Rovis T. (2021). ACS Catal..

[cit25] Rohe S., Morris A. O., McCallum T., Barriault L. (2018). Angew. Chem., Int. Ed..

[cit26] Deng H. P., Zhou Q., Wu J. (2018). Angew. Chem., Int. Ed..

[cit27] Deng H. P., Fan X. Z., Chen Z. H., Xu Q. H., Wu J. (2017). J. Am. Chem. Soc..

[cit28] Zidan M., Morris A. O., McCallum T., Barriault L. (2020). Eur. J. Org. Chem..

[cit29] Nielsen M. K., Shields B. J., Liu J., Williams M. J., Zacuto M. J., Doyle A. G. (2017). Angew. Chem., Int. Ed..

[cit30] Ackerman L. K. G., Alvarado J. I. M., Doyle A. G. (2018). J. Am. Chem. Soc..

[cit31] Yang Q., Wang Y. H., Qiao Y., Gau M., Carroll P. J., Walsh P. J., Schelter E. J. (2021). Science.

[cit32] Hu A., Guo J. J., Pan H., Zuo Z. (2018). Science.

[cit33] Xu Y., Noirbent G., Brunel D., Liu F., Gigmes D., Sun K., Zhang Y., Liu S., Morlet-Savary F., Xiao P., Dumur F., Lalevée J. (2020). Eur. Polym. J..

[cit34] Dadashi-Silab S., Doran S., Yagci Y. (2016). Chem. Rev..

[cit35] Block H., Ledwith A., Taylor A. R. (1971). Polymer.

[cit36] Amirzadeh G., Schnabel W. (1981). Makromol. Chem..

[cit37] Kowalska A., Sokolowski J., Bociong K. (2021). Polymers.

[cit38] Meinwald J., Klingele H. O. (1966). J. Am. Chem. Soc..

[cit39] Monroe B. M., Weiner S. A. (1969). J. Am. Chem. Soc..

[cit40] Maruyama K., Takahashi T. (1974). Chem. Lett..

[cit41] Taskin O. S., Yilmaz G., Tasdelen M. A., Yagci Y. (2014). Polym. Int..

[cit42] Pyszka I., Kucybała Z., Pączkowski J. (2004). Macromol. Chem. Phys..

[cit43] Tripathi C. B., Ohtani T., Corbett M. T., Ooi T. (2017). Chem. Sci..

[cit44] Mateos J., Cuadros S., Vega-Peñaloza A., Dell’Amico L. (2021). Synlett.

[cit45] Ravelli D., Fagnoni M., Albini A. (2013). Chem. Soc. Rev..

[cit46] Hunt A. J. , Farmer T. J. and Clark J. H., Element Recovery and Sustainability, The Royal Society of Chemistry, Lindon, 2013, pp. 1–28

[cit47] Anastas P., Eghbali N. (2010). Chem. Soc. Rev..

[cit48] Anastas P., Kirchhoff M. M. (2002). Acc. Chem. Res..

[cit49] Li C. J., Trost B. M. (2008). Proc. Natl. Acad. Sci. U.S.A..

[cit50] Garrett C. E., Prasad K. (2004). Adv. Synth. Catal..

[cit51] Ruccolo S., Qin Y., Schnedermann C., Nocera D. G. (2018). J. Am. Chem. Soc..

[cit52] Allonas X., Fouassier J.-P., Angiolini L., Caretti D. (2001). Helv. Chim. Acta.

[cit53] Singh A., Scott A. R., Sopchyshyn F. (1969). J. Phys. Chem..

[cit54] Hodgkiss J. M. , Rosenthal J. and Nocera D. G., Handbook of Hydrogen Transfer. Physical and Chemical Aspects of Hydrogen Transfer, ed. J. T. Hynes, J. P. Klinman, H.-H. Limbach and R. L. Schowen, Wiley–VCH: Weinheim, Germany, 2006, vol. II part IV, ch. 17, pp. 503–562

[cit55] Land E. J., Ebert M. (1967). Trans. Faraday Soc..

[cit56] Larson D. B., Arnett J. F., Wahlborg A., McGlynn S. P. (1974). J. Am. Chem. Soc..

[cit57] Lias S. G. , Bartmess J. E., Liebman J. F., Holmes J. L., Levin R. D. and Mallard W. G., NIST Chemistry WebBook, NIST Standard Reference Database, ed. P. J. Linstrom and W. G. Mallard, National Institute of Standards and Technology, Gaithersburg MD, 20899, (retrieved November 3, 2023)

[cit58] Rieth A. J., Qin Y., Martindale B. C. M., Nocera D. G. (2021). J. Am. Chem. Soc..

[cit59] Berg N., Bergwinkl S., Nuernberger P., Horinek D., Gschwind R. M. (2021). J. Am. Chem. Soc..

[cit60] You W., Ganley J. M., Ernst B. G., Peltier C. R., Ko H. Y., DiStasio R. A., Knowles R. R., Coates G. W. (2021). Chem. Sci..

[cit61] Huang C. Y., Li J., Liu W., Li C. J. (2019). Chem. Sci..

[cit62] Proctor R. S. J., Chuentragool P., Colgan A. C., Phipps R. J. (2021). J. Am. Chem. Soc..

[cit63] Dantas J. A., Echemendía R., Santos M. S., Paixão M. W., Ferreira M. A. B., Corrêa A. G. (2020). J. Org. Chem..

[cit64] Campbell M. W., Yuan M., Polites V. C., Gutierrez O., Molander G. A. (2021). J. Am. Chem. Soc..

[cit65] Han L., Xia J. B., You L., Chen C. (2017). Tetrahedron.

[cit66] Capaldo L., Ravelli D. (2017). Eur. J. Org. Chem..

[cit67] Miyaura N., Suzuki A. (1995). Chem. Rev..

[cit68] Molander G. A., Biolatto B. (2002). Org. Lett..

[cit69] Barder T. E., Buchwald S. L. (2004). Org. Lett..

[cit70] Kowalska A., Sokolowski J., Bociong K. (2021). Polymers.

