# Supplementary material for: Chemoselective bond activation by unidirectional and asynchronous PCET using ketone photoredox catalysts
Source: Chem Sci. 2023 Nov 2;14(47):13776–82. doi: 10.1039/d3sc04362b (PMC10699566; doi:10.1039/d3sc04362b)
Supplement: SC-014-D3SC04362B-s001 [file SC-014-D3SC04362B-s001.pdf]

Electronic Supplementary Material (ESI) for Chemical Science.
This journal is © The Royal Society of Chemistry 2023

Supporting Information

Chemoselective Bond Activation by Unidirectional and Asynchronous PCET Using Ketone Photoredox Catalysts

Rui Sun,[‡] Serge Ruccolo,[‡, ¥] Daniel Luis do Nascimento,[‡] Yangzhong Qin, [¥] Nathaniel Hibbert, and Daniel G. Nocera*

Department of Chemistry and Chemical Biology, Harvard University, 12 Oxford Street, Cambridge, Massachusetts 02138, USA

Email: dnocera@fas.harvard.edu

Table of Contents

A. General Considerations S3

B. Synthesis of New Amide Precursors and Products S3

Figure S1. NMR spectra for compound **13** S6

Figure S2. NMR spectra for compound **8** S7

Figure S3. NMR spectra for compound **14** S8

Figure S4. NMR spectra for compound **12** S9

Figure S5. Crude NOESY spectra of **10** and **11** S10

Figure S6. NMR spectra for compound **15** S11

Figure S7. IR spectra for proteo- and deutero-**15** S11

C. Cyclic Voltammetry and Spectroelectrochemistry S12

Figure S8. CV and spectroelectrochemistry of **CQ** S12

D. Single-Wavelength Kinetic Studies and Transient Absorption Spectroscopy S12

Figure S9. TA spectra of **CQ** with phenol in DCM S13

Figure S10. TA kinetic trace of **CQ** with substrate **1′** in DCM S13

E. NMR Study of the Ground-State Association Between **CQ** and **1** S14

Figure S11. ^{1}H NMR study of association between **CQ** and **1** S14

F. Steady-State Stern-Volmer Studies S15

Table S1. Quenching rates (k_q) of ***CQ** with different quenchers S15

Figure S12. Stern-Volmer plots for different quenchers reacting with **CQ** S16

G. Photochemical CQ and Ketone-Mediated Intramolecular Hydroamidation S17

Figure S13. Time traces for the yields of cyclized product S17

Figure S14. Cycloamidation yields with various ketones S18

H. Reaction Quantum Yield Measurements S18

I. References S20

A. General Considerations

All manipulations were performed with the rigorous exclusion of air and moisture unless otherwise stated. Commercial reagents were stored in a N_2-filled glovebox and used without further purification. All liquid reagents and deuterated solvents were degassed by three cycles of freeze-pump-thaw and stored over activated 3Å molecular sieves prior to use. All non-deuterated solvents were purified by the method of Grubbs and stored over activated 3Å molecular sieves.[1] Camphorquinone, tributylmethylammonium dibutyl phosphate, triethylamine, tetrabutyl-ammonium chloride (TBACl) and potassium hydrogen fluoride (KHF_2) were purchased from Sigma Aldrich. The Ir photooxidant, (2,2′-bipyridine)bis[3,5-difluoro-2-[5-trifluoromethyl-2-pyridinyl-kN)phenyl-kC]iridium(III) hexafluorophosphate, was purchased from Strem Chemicals. NMR spectra were recorded at the Laukien-Purcell Instrumentation Center in the Department of Chemistry and Chemical Biology at Harvard University on an Agilent DD2 spectrometer operating at 600 MHz, a Varian Unity/Inova spectrometer operating at 500 MHz, or a JEOL ECZ400S spectrometer operating at 400 MHz. Chemical shifts are reported in ppm and referenced to the residual proton or carbon signals of the solvent. For ^{19}F NMR, hexafluorobenzene at –164.9 ppm was used as external standard. MALDI mass spectra were collected on a Bruker UltrafleXtreme MALDI-TOF/TOF mass spectrometer. Solutions of matrix (pyrene) and analyte (20:1) were prepared such that the final concentration of matrix and analyte was ca. 100 mM and 5 mM, respectively. A 1 μL aliquot of the mixed solution was spotted on the MALDI target plate and allowed to evaporate by the dried-droplet method. Spectra were calibrated internally with pyrene (*m/z* 202.078).

B. Synthesis of New Amide Precursors and Products

The known amide substrates were either purchased or prepared as previously described,[2] whereas the new ones were synthesized according to the procedures reported below.

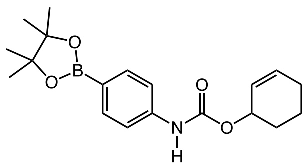

Cyclohex-2-en-1-yl (4-(Bpin)phenyl)carbamate (13). In a 20 mL scintillation vial equipped with a PTFE-coated stir bar, 2-cyclohexen-1-ol (0.404 g, 4.08 mmol, 1.00 equiv) and triethylamine (1.75 mL, 12.7 mmol, 3.20 equiv) were combined and CH_2Cl_2 was added (2 mL). 4-Isocyanatobenzeneboronic acid pinacol ester (1.00 g, 4.11 mmol, 1.00 equiv) was added as a solid and more CH_2Cl_2 (3 mL) was used to effect quantitative transfer. After stirring the yellow solution at room temperature for 18 h, an aliquot was removed, dried, and subjected to ^1H NMR analysis, which showed complete consumption of the starting materials. The sample was brought back and recombined with the reaction. Volatiles were removed from the solution *in vacuo* and the residual solid was redissolved with minimum CH_2Cl_2 and subjected to a chromatographic column (0 → 30% EtOAc in hexanes; the desired product elutes first). After removing the solvents *in vacuo* a white solid remained and was dried for 18 h. Yield after drying: 0.360 g (26%). ^1H NMR ($CDCl_3$, 400 MHz): δ 7.75 (d, J = 8.5 Hz, 2H), 7.38 (d, J = 8.1 Hz, 2H), 6.63 (m, 1H), 5.99 (m, 1H), 5.78 (s, 1H), 5.28 (s, 1H), 2.22 – 1.59 (m, 6H), 1.33 (s, 12H). $^{13}C\{^1H\}$ NMR ($CDCl_3$, 101 MHz): δ 153.06, 140.86, 136.03, 133.13, 125.79, 117.44, 83.81, 69.08, 28.64, 25.03, 25.01, 18.89. See Figure S1 for spectra. MALDI-TOF MS, *m/z*: 344.205 (Calcd: 344.203 for $C_{19}H_{27}B_1N_1O_4^+$).

3-(4-(Bpin)phenyl)hexahydrobenzo[d]oxazol-2(3H)-one (8). In the glovebox, compound **13** (0.176 g, 0.518 mmol, 1.00 equiv), camphorquinone (17.6 mg, 0.106 mmol, 0.200 equiv) and phenyl disulfide (12.4 mg, 0.057 mmol, 0.100 equiv) were combined as solids in a 20 mL scintillation vial containing a PTFE-coated stir bar. CH_2Cl_2 (5 mL) was added, the vial was capped, and sealed with electrical tape. The reaction was then brought outside the glovebox and irradiated using a Kessil A160WE Tuna Blue LED lamp under fan cooling. After 24 h, the reaction was brought back to the glovebox and an aliquot was retrieved for NMR analysis, which showed still the presence of starting material. More camphorquinone (13.0 mg, 0.08 mmol, 0.20 equiv) and phenyl disulfide (12.0 mg, 0.06 mmol, 0.10 equiv) were added to the reaction, which was stirred under blue LED for another 14 h. After that, another aliquot was retrieved and analyzed by ^{1}H NMR, which showed complete consumption of the starting material. The reaction had its volatiles removed and the residue was redissolved with minimum CH_2Cl_2. After that, the crude material was subjected to a chromatographic column (100% hexanes, 1 CV; 0 → 70% EtOAc in hexanes, 10 CV; 70% EtOAc, 2 CV). The volatiles were removed under reduced pressure, yielding a faint-yellow solid, which was further dried for 18 h. Yield after drying: 0.140 g (80%). ^{1}H NMR ($CDCl_3$, 400 MHz): δ 7.80 (d, J = 8.5 Hz, 2H), 7.53 (d, J = 8.6 Hz, 2H), 4.66 (s, 1H), 4.31 (s, 1H), 1.34 (m, 20H). ^{13}C{^{1}H} NMR ($CDCl_3$, 101 MHz): δ 155.56, 140.10, 135.97, 119.11, 83.93, 73.35, 55.79, 26.80, 26.43, 25.01, 24.98, 20.43, 19.33. See Figure S2 for spectra. MALDI-TOF MS, *m/z*: 344.192 (Calcd: 344.203 for $C_{19}H_{27}B_1N_1O_4^+$).

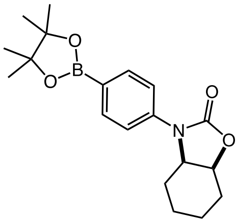

Tetrabutylammonium (4-(((cyclohex-2-en-1-yloxy)carbonyl)amino)-phenyl)trifluoroborate (14). A 50 mL Schlenk round-bottom flask equipped with a PTFE-coated stir bar was charged with compound **13** (0.202 g, 0.589 mmol, 1.00 equiv) and dissolved with MeOH (15 mL). After cooling to 0°C, a suspension of KHF_2 in MeOH (10 mL) was transferred to the stirred solution. After 5 min, the reaction was removed from the ice bath and allowed to stir at room temperature for 2 h. After that, the volatiles were evaporated using a rotavap. To help remove most of the pinacol side-product, the crude material was redissolved with MeOH (10 mL) and water (5 mL) and the volatiles were evaporated. This process was repeated once more. To the resulting material, acetone (10 mL) was added to create a white cloudy suspension, which was stirred for 30 min. After that, the reaction was filtered through a PTFE filter (0.45 μm) into a 20 mL scintillation vial. To the stirring colorless solution, tetrabutylammonium chloride was added (0.166 g, 0.597 mmol, 1.00 equiv), immediately forming a white precipitate. The reaction was stirred at room temperature for 30 min, after which it was filtered through a small pad of silica. The silica was further washed with acetone (ca. 3 mL) and the resulting filtrate had its volatiles removed in the rotavap, resulting in a sticky colorless oil. Et_2O (5 mL) was added to the crude material and stirred vigorously for 5 min. The supernatant was carefully removed with a pipette and the process was repeated once more with hexanes (5 mL). After pulling vacuum, a white solid remained, which was dried for a further 18 h. Yield after drying: 0.210g (68%). ^{1}H NMR ($CDCl_3$, 400 MHz): δ 7.54 (d, J = 8.1 Hz, 2H), 7.20 (d, J = 7.8 Hz, 2H), 6.52 (s, 1H), 6.02 – 5.88 (m, 1H), 5.84 – 5.70 (m, 1H), 5.23 (s, 1H), 3.19 – 3.05 (m, 8H), 2.15 – 1.31 (m, 22H), 0.97 (t, J = 7.3 Hz, 12H). ^{13}C{^{1}H} NMR ($CDCl_3$, 101 MHz): δ 135.65, 132.69, 132.56, 126.21, 117.84, 68.38, 58.69, 28.73, 25.04, 24.01, 19.76, 18.97,

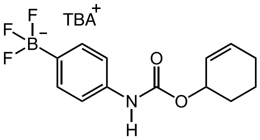

13.78. $^{19}F\{^1H\}$ NMR ($CDCl_3$, 376 MHz): – 139.66. See Figure S3 for spectra. ESI-TOF MS, *m/z*: 284.108 (Calcd: 284.108 for $C_{13}H_{14}BF_3NO_2^-$).

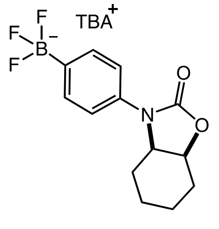

Tetrabutylammonium trifluoro(4-(2-oxohexahydrobenzo[d]oxazol-3(2H)-yl)phenyl)borate (12). In the glovebox, compound **14** (0.206 g, 0.391 mmol, 1.00 equiv), camphorquinone (19.4 mg, 0.177 mmol, 0.500 equiv) and phenyl disulfide (16.0 mg, 0.073 mmol, 0.200 equiv) were combined as solids in a 20 mL scintillation vial containing a PTFE-coated stir bar. CH_2Cl_2 (5 mL) was added, the vial was capped, and sealed with electrical tape. The reaction was brought outside the glovebox and irradiated using a Kessil A160WE Tuna Blue LED lamp under fan cooling. After 24 h, the reaction was brought back into the glovebox and an aliquot was retrieved for NMR analysis, which showed complete consumption of the starting material. The reaction had its volatiles removed, CH_2Cl_2 (2 mL) and hexanes (5 mL) were added and the reaction was stirred vigorously for ca. 5 min, after which the supernatant was removed. To the crude residue, CH_2Cl_2 (3 mL) was added and the suspension was filtered through a silica plug. After washing the silica with more CH_2Cl_2 (3 mL), hexanes (3 mL) was added to the filtrate and the solution was stirred for 5 min. Then, the supernatant was removed and the orange sticky solid was dried for 18 h. Yield after drying: 0.150g (73%). ^1H NMR ($CDCl_3$, 400 MHz): δ 7.62 (d, J = 8.1 Hz, 2H), 7.18 (d, J = 7.9 Hz, 2H), 4.62 (m, 1H), 4.20 (q, J = 6.2 Hz, 1H), 3.03 – 2.93 (m, 8H), 2.04 – 1.22 (m, 24H), 0.95 (t, J = 7.2 Hz, 12H). $^{13}C\{^1H\}$ NMR ($CDCl_3$, 101 MHz): δ 156.83, 134.47, 132.73, 121.79, 73.46, 58.44, 56.71, 27.15, 26.01, 23.83, 19.89, 19.69, 13.75. $^{19}F\{^1H\}$ NMR ($CDCl_3$, 376 MHz): – 140.23 See Figure S4 for spectra. ESI-TOF MS, *m/z*: 284.107(Calcd: 284.108 for $C_{13}H_{14}BF_3NO_2^-$).

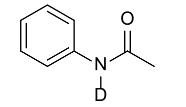

***N*-Phenylacetamide-*N*-d (15).** In a N_2-filled glovebox, potassium *tert*-butoxide (0.187 g, 1.66 mmol, 1.50 equiv) was dissolved in diethyl ether (3 mL) and added to a suspension of acetanilide (0.150 g, 1.11 mmol, 1.00 equiv) in diethyl ether (10 mL). The cloudy mixture was stirred overnight and the solid was collected by filtration, washed with diethyl ether (5 × 5 mL), and dried *in vacuo*. Heavy water (5 mL) was then added to the solid and the mixture was sonicated for 30 min. The cloudy suspension was then extracted with DCM-d_2 (2 × 3 mL), and the organic phase was dried over anhydrous Na_2SO_4. The solvent was removed *in vacuo* to obtain the titular compound as a white solid. ^1H NMR ($CDCl_3$, 400 MHz): δ 7.50 (d, J = 7.9 Hz, 2H), 7.32 (t, J = 7.8 Hz, 2H), 7.11 (t, J = 7.9 Hz, 1H), 2.18 (s, 3H). See Figure S6 for the ^1H NMR spectrum and a comparison with the ^1H NMR spectrum of proteo-acetanilide showing the disappearance of the amide N–H resonance. ESI-TOF MS, *m/z*: 137.0818 (calculated: 137.0820 for $C_8H_9DNO^+$). See Figure S7 for a comparison of the IR spectra between proteo-acetanilide and *N*-phenylacetamide-*N*-d.

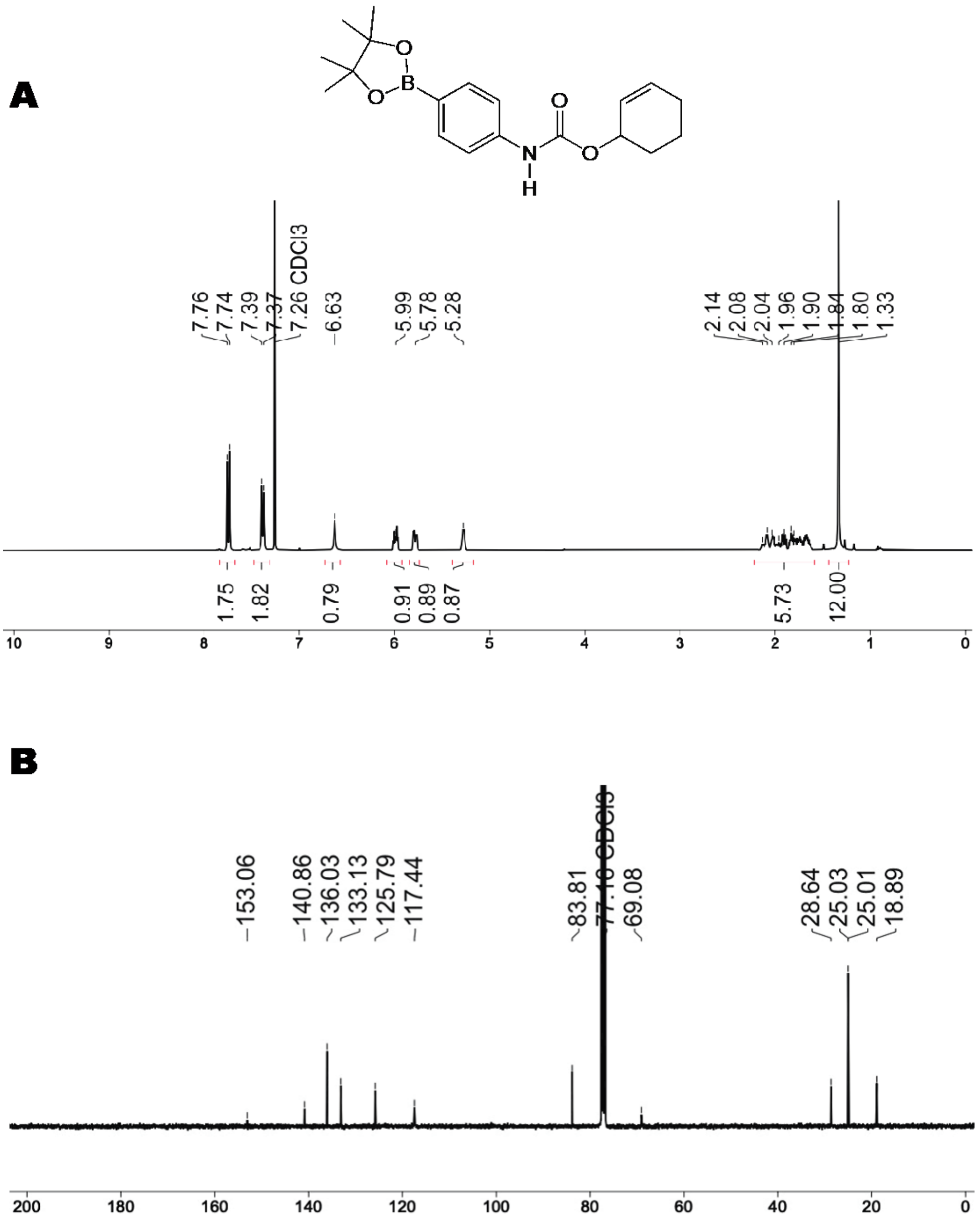

Figure S1. NMR spectra for compound **13** in $CDCl_3$. (**A**) ^{1}H NMR (400 MHz) and (**B**) $^{13}C\{^{1}H\}$ NMR (101 MHz).

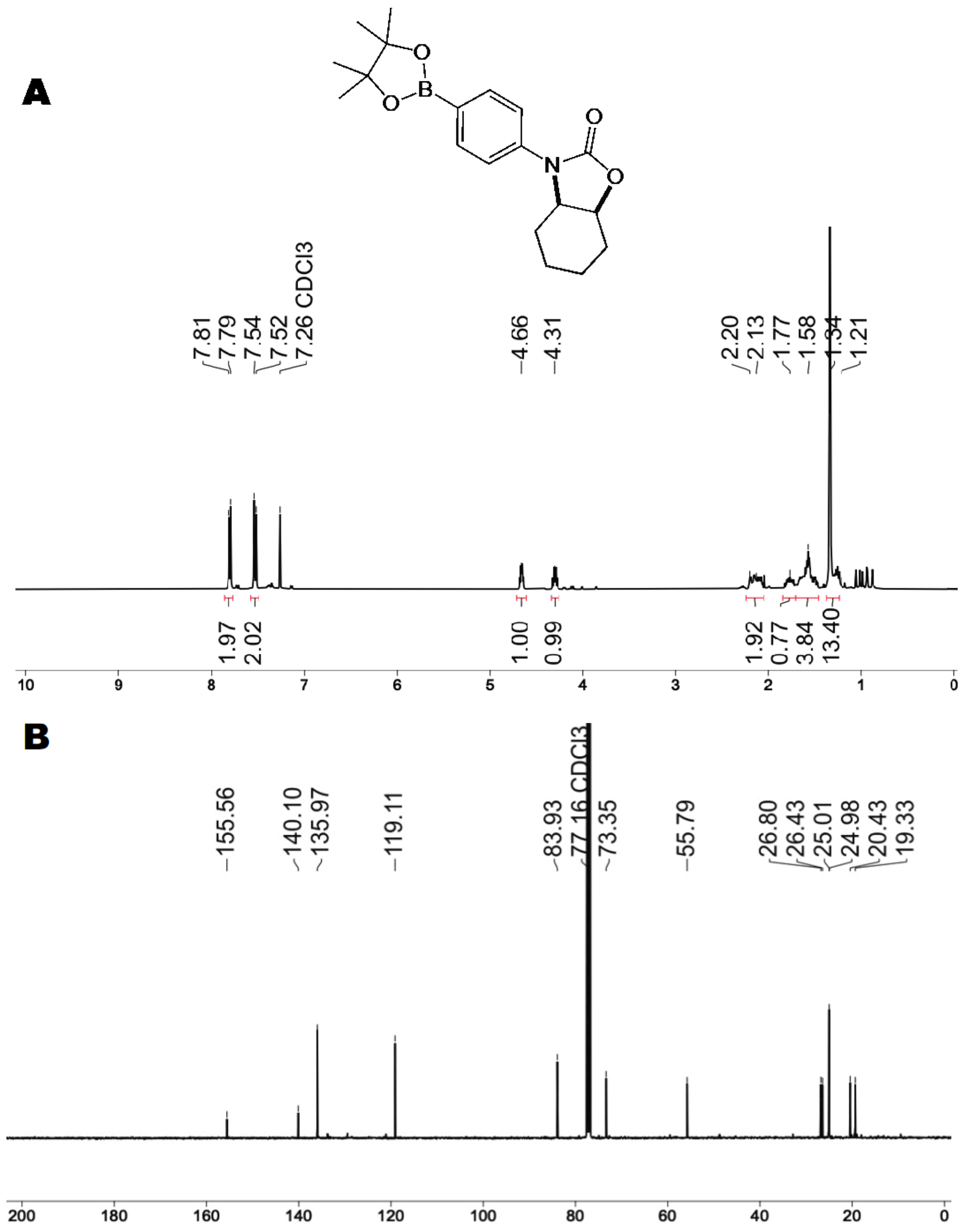

Figure S2. NMR spectra for compound **8** in $CDCl_3$. (**A**) ^{1}H NMR (400 MHz) and (**B**) $^{13}C\{^{1}H\}$ NMR (101 MHz).

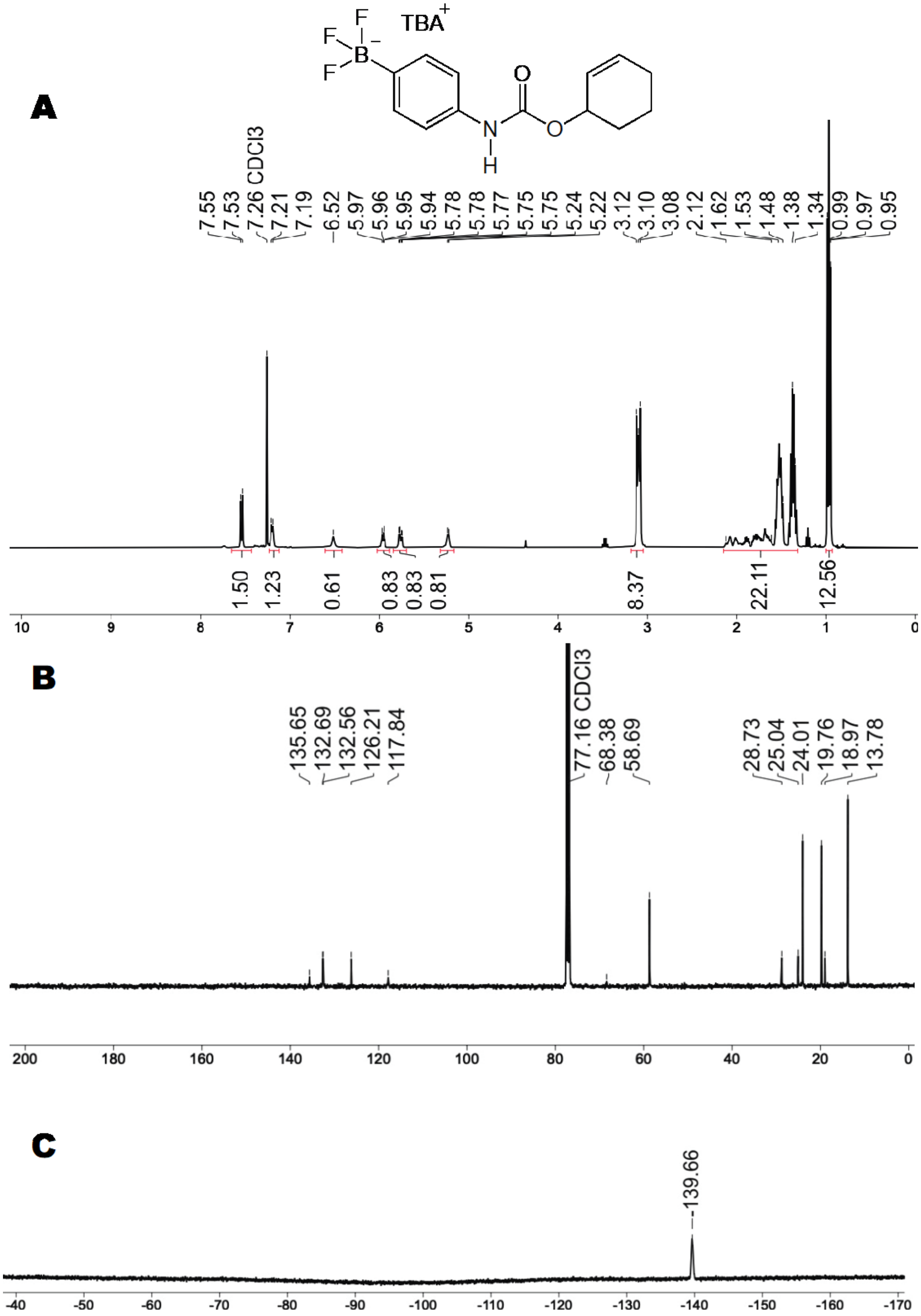

Figure S3. NMR spectra for compound **14** in $CDCl_3$. (**A**) ^{1}H NMR (400 MHz), (**B**) $^{13}C\{^{1}H\}$ NMR (101 MHz) and (**C**) $^{19}F\{^{1}H\}$ NMR (376 MHz).

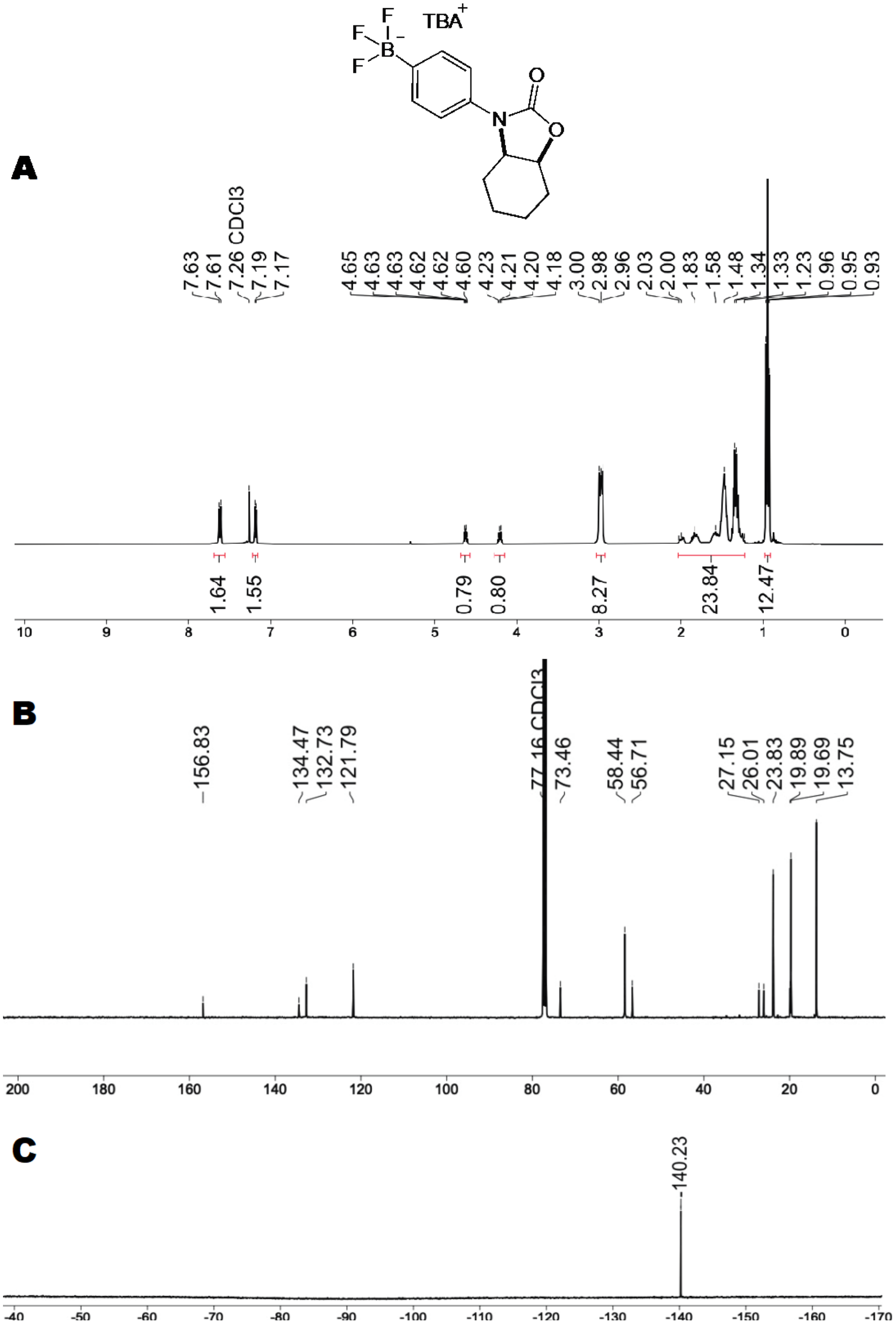

Figure S4. NMR spectra for compound **12** in $CDCl_3$. (**A**) ^{1}H NMR (400 MHz). (**B**) $^{13}C\{^{1}H\}$ NMR (101 MHz) and (**C**) $^{19}F\{^{1}H\}$ NMR (376 MHz).

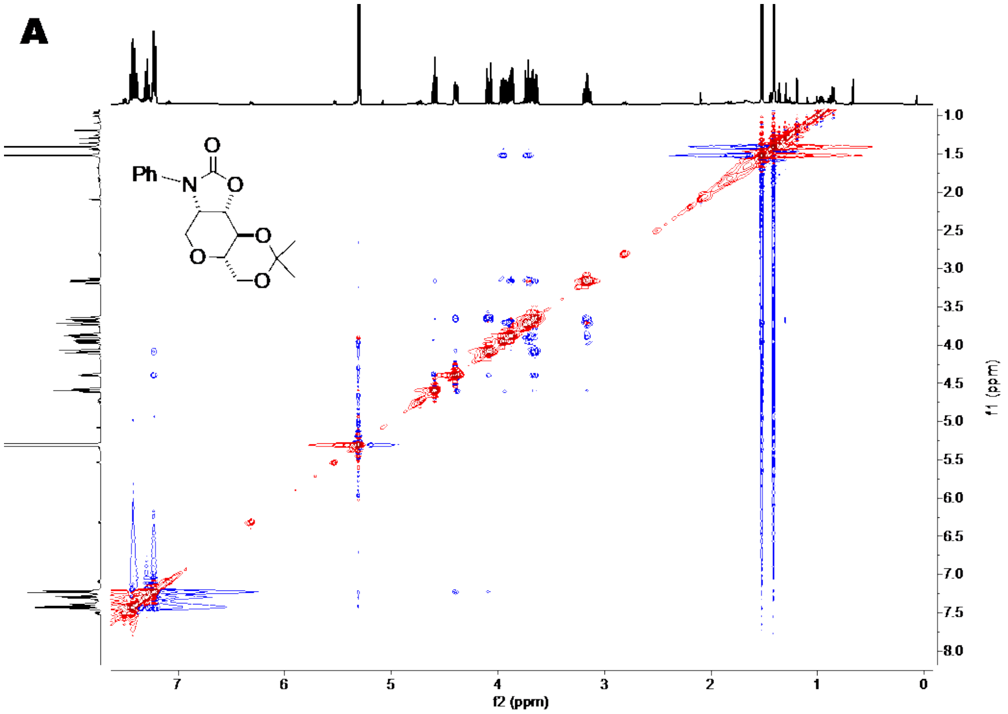

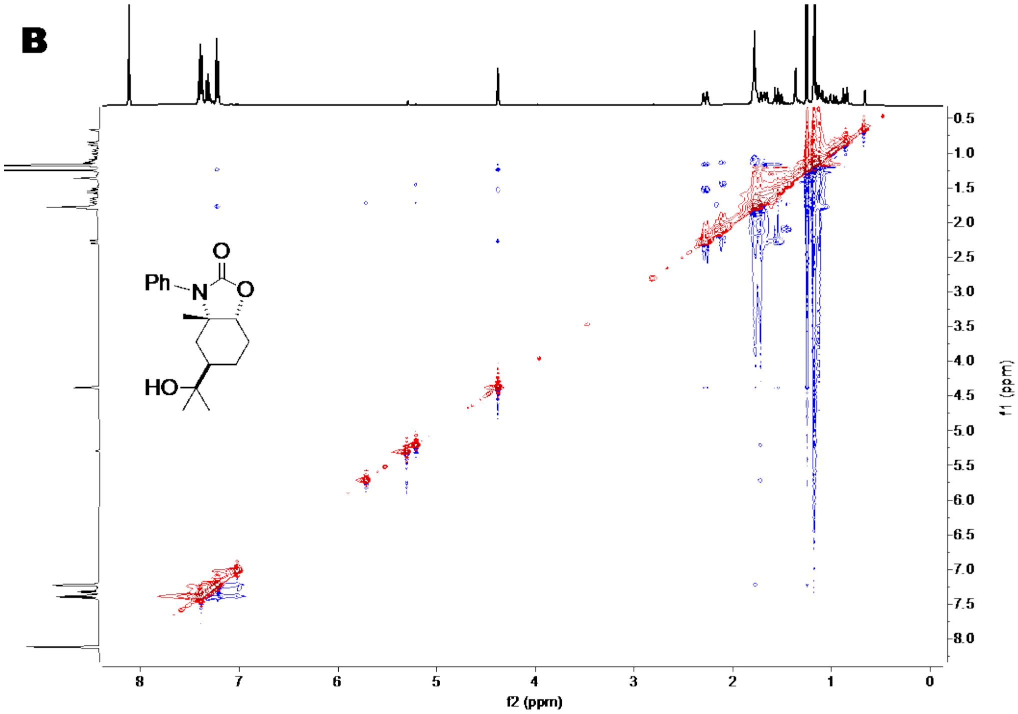

Figure S5. Crude 2D NOESY NMR (400 MHz, CD_2Cl_2) spectra of **10** and **11**. Both (**A**) and (**B**) agree with literature precedent.[2]

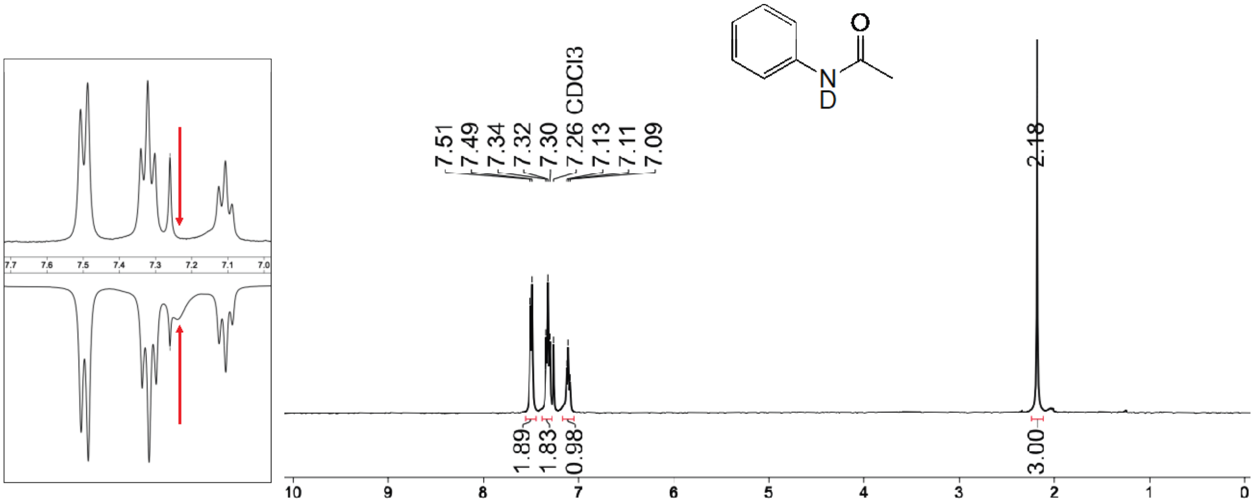

Figure S6. ^{1}H NMR (400 MHz, $CDCl_3$) spectrum for *N*-phenylacetamide-*N*-d (**15**). Inset shows the aromatic region for protic (bottom) vs deuterated (top) compounds. Red arrows indicate the disappearance of the N–H signal in the deuterated version.

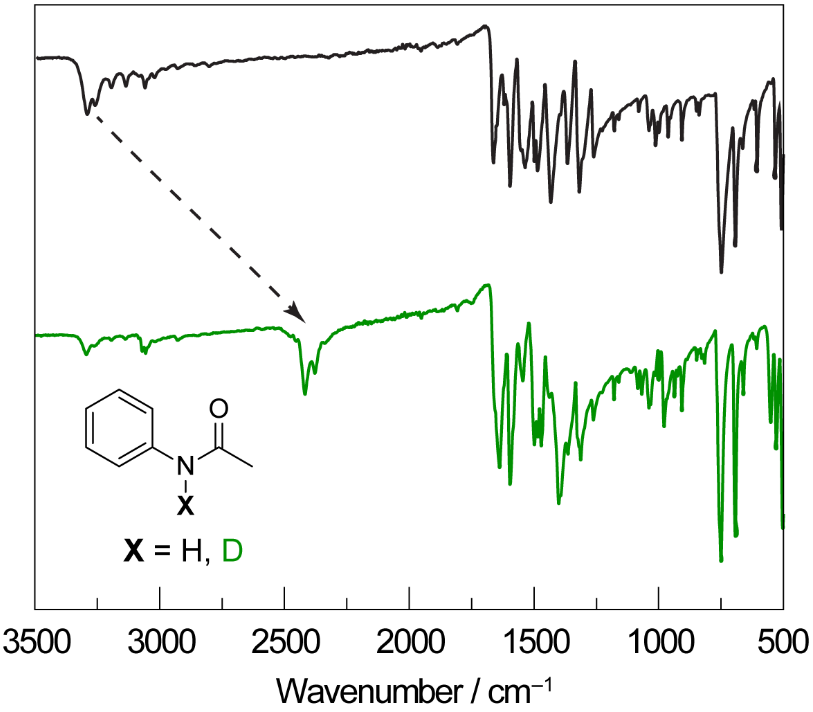

Figure S7. Comparison of the IR spectra for proteo-acetanilide (▬ black trace) and *N*-phenylacetamide-*N*-d (▬ green trace) showing a redshift of the N–D stretching frequency relative to the N–H stretching frequency.

C. Cyclic Voltammetry and Spectroelectrochemistry

All electrochemical experiments were performed with a CH Instruments 760D Electrochemical Workstation (Austin, Texas) and CHI Version 10.03 software in a N_2-filled glovebox. **CQ** was dissolved in an electrolyte solution containing 0.1 M n-Bu_4NBF_4 in DCM. A three-electrode undivided cell configuration with a glassy carbon working electrode, Pt wire counter electrode, and non-aqueous Ag^+/Ag reference electrode was used for all cyclic voltammetry (CV) experiments. Working electrodes were sequentially polished on felt using diamond pastes of 3 μm and 1 μm before use. Ferrocene (Fc) was added to each sample at the end of each measurement. Spectroelectrochemical measurements were performed using a 0.5 mm thin-layer quartz cuvette with a Pt mesh working electrode, non-aqueous Ag^+/Ag reference electrode, and Pt wire counter electrode. The UV-vis absorption spectra were recorded with OceanView 1.4.1 coupled with a light source (Ocean Optics DT-MINI-2GS) and spectrometer (Ocean Optics, USB4000).

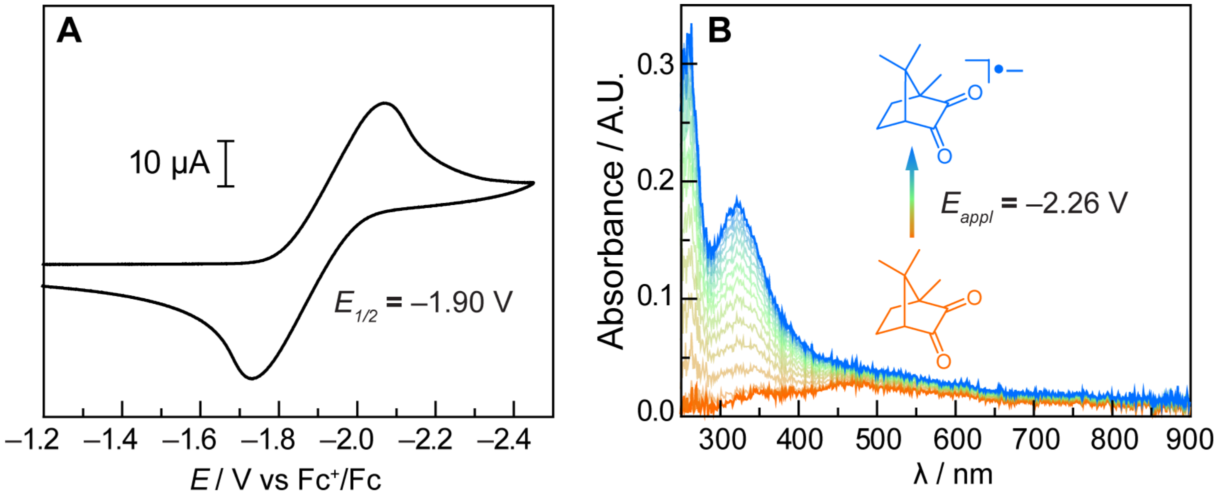

Figure S8. Electrochemical studies on **CQ**. (**A**) Cyclic voltammogram of 2 mM **CQ** in DCM with 0.1 M [TBA][PF_6] as the supporting electrolyte. (**B**) Spectroelectrochemistry on 2 mM **CQ** in DCM with 0.1 M [TBA][PF_6] as the supporting electrolyte in a 0.5 mm pathlength cell using a Pt mesh working electrode.

D. Single-Wavelength Kinetic Studies and Transient Absorption Spectroscopy

The nanosecond transient absorption (TA) spectroscopy setup was described previously in detail.[3] A Quanta-Ray Nd:YAG laser (SpectraPhysics) provides 3rd harmonic laser pulses at 355 nm with a repetition rate of 10 Hz and pulse width of ~10 ns (FWHM). A MOPO (SpectraPhysics) was used to provide tunable laser pulses in the visible region. Typical excitation energy was adjusted to ~4 mJ/pulse @460 nm. Solutions were prepared in the glovebox and placed through a 1.0 cm flow cell (Starna) with a peristaltic pump for spectral acquisition. To extract the rate constants for HAT (k_H) and back reaction (k_{BR}), we use the following rate equation to model the TA trace:

$$\frac{d[\mathbf{1'}\bullet]}{dt} = k_H[\mathbf{1'}] - k_{BR}[\mathbf{1'}\bullet][\mathrm{CQH}\bullet]$$

As shown in Figure 4 (A and B) of the main text, the signal at 430 nm is due to the amidyl radical exclusively,[4] therefore, the signal can be written as $S_{430nm} = \varepsilon[\mathbf{1'}\bullet]$ where $\varepsilon = 4100\ M^{-1}\ cm^{-1}$ is the extinction coefficient of the amidyl radical at 430 nm, determined from previous studies.[4]

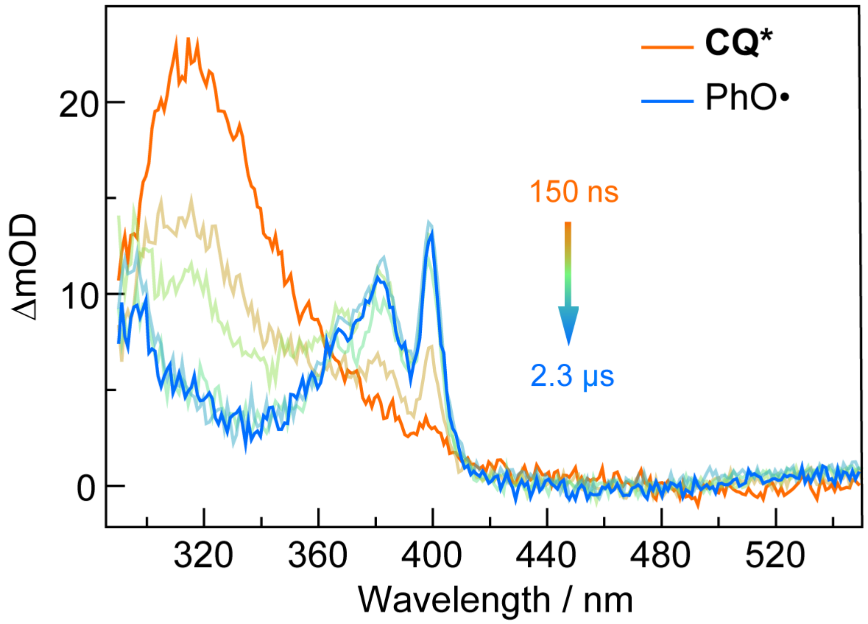

Figure S9. TA spectra of **CQ** (10 mM) and phenol (20 mM) in DCM showing the evolution from an initial spectrum dominated by **CQ*** (▬ orange trace) to one dominated by PhO• (▬ blue trace). λ_{exc} = 460 nm.

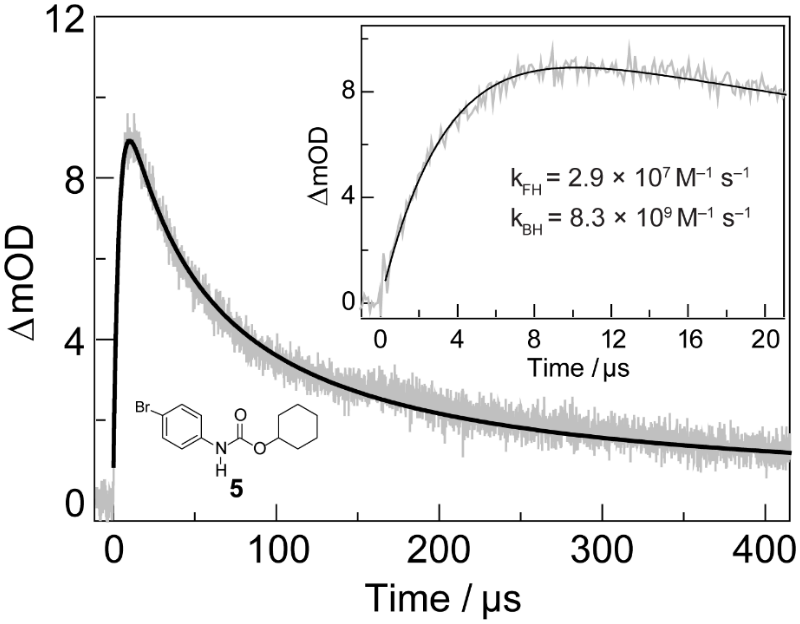

Figure S10. Kinetic trace monitored at 430 nm of 5 mM **CQ** and 10 mM amide substrate **1′** in DCM. Inset shows a magnified view at shorter timescales along with extracted rate constants. λ_{exc} = 460 nm.

E. NMR Study of the Ground-State Association Between CQ and 1

Solutions of amide **1** (2 mM) and varying amounts of **CQ** (0, 20, 30, 40, and 50 mM) were prepared in anhydrous DCM-d_2. The association constant (K_a) between **CQ** and **1** in DCM-d_2 was determined using ^{1}H NMR spectroscopy by plotting [CQ]/Δδ against [CQ] and calculating K_a = slope/intercept, where $\Delta\delta = \delta_1 - \delta_{obs}$ is the difference in chemical shifts of the N-H proton of **1** by itself (δ_1) and **1** in the presence of added **CQ** (δ_{obs}).[4, 5]

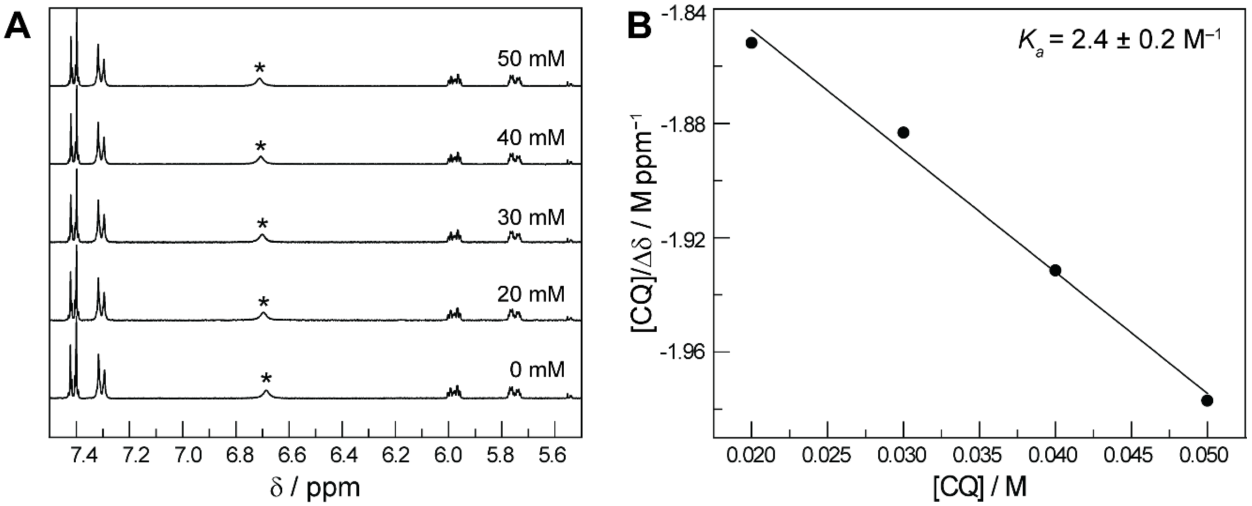

Figure S11. ^{1}H NMR study of association between amide **1** and **CQ**. (**A**) Stacked ^{1}H NMR spectra showing the change in the amide N-H signal of **1** (marked by *) with varying concentrations of added **CQ**. (**B**) Plot of [**CQ**]/Δδ against [**CQ**] for solutions of **1** with varying amounts of **CQ** (black circle) and linear fit (solid line).

F. Steady-State Stern-Volmer Studies

Fluorescence was monitored on a QM4 fluorometer (Photon Technology International). Different samples were obtained by sequentially diluting a stock solution of the quencher and photocatalyst with a solution containing only the photocatalyst and transferred into 1 cm quartz cuvettes (Starna) for measurement. Steady-state quenching studies were performed by using the peak phosphorescence intensity with excitation at 450 nm. Samples were exposed to air after the measurements in order to fully quench the phosphorescence. The resulting fluorescence spectrum was subtracted from the total emission spectra in order to obtain the phosphorescence-only spectra.

Table S1. Correlation of the quenching rate (k_q) of ***CQ** in DCM with different thermodynamic parameters of the quenchers.

	**Ionization Energy (eV)**[6]	k_q **(M^{-1} s^{-1})**[a]	**X–H BDE (kcal/mol)**[7]	**pK_a in DMSO**[8]
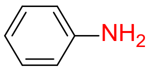	7.7	1.12 (0.08) × 10^{11}	90 (gas phase)	31
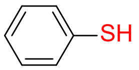	8.3	3.11 (0.14) × 10^{9}	84 (TR-PAC)	10
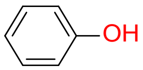	8.5	3.18 (0.14) × 10^{9}	88 (gas phase)	18
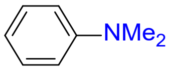	7.1	2.75 (0.11) × 10^{10}	–	–
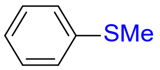	7.9	4.51 (0.29) × 10^{7}	–	–
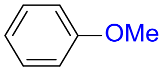	8.2	5.75 (0.39) × 10^{4}	–	–
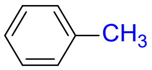	8.8	*not observed*	90 (gas phase)	43

[a] Calculated from the Stern-Volmer constant (K_{SV}) using a value of τ = 30.6 (0.1) µs for the lifetime of the **CQ** triplet state, as determined from time-resolved emission spectroscopy (see Figure S12 for Stern-Volmer plots).

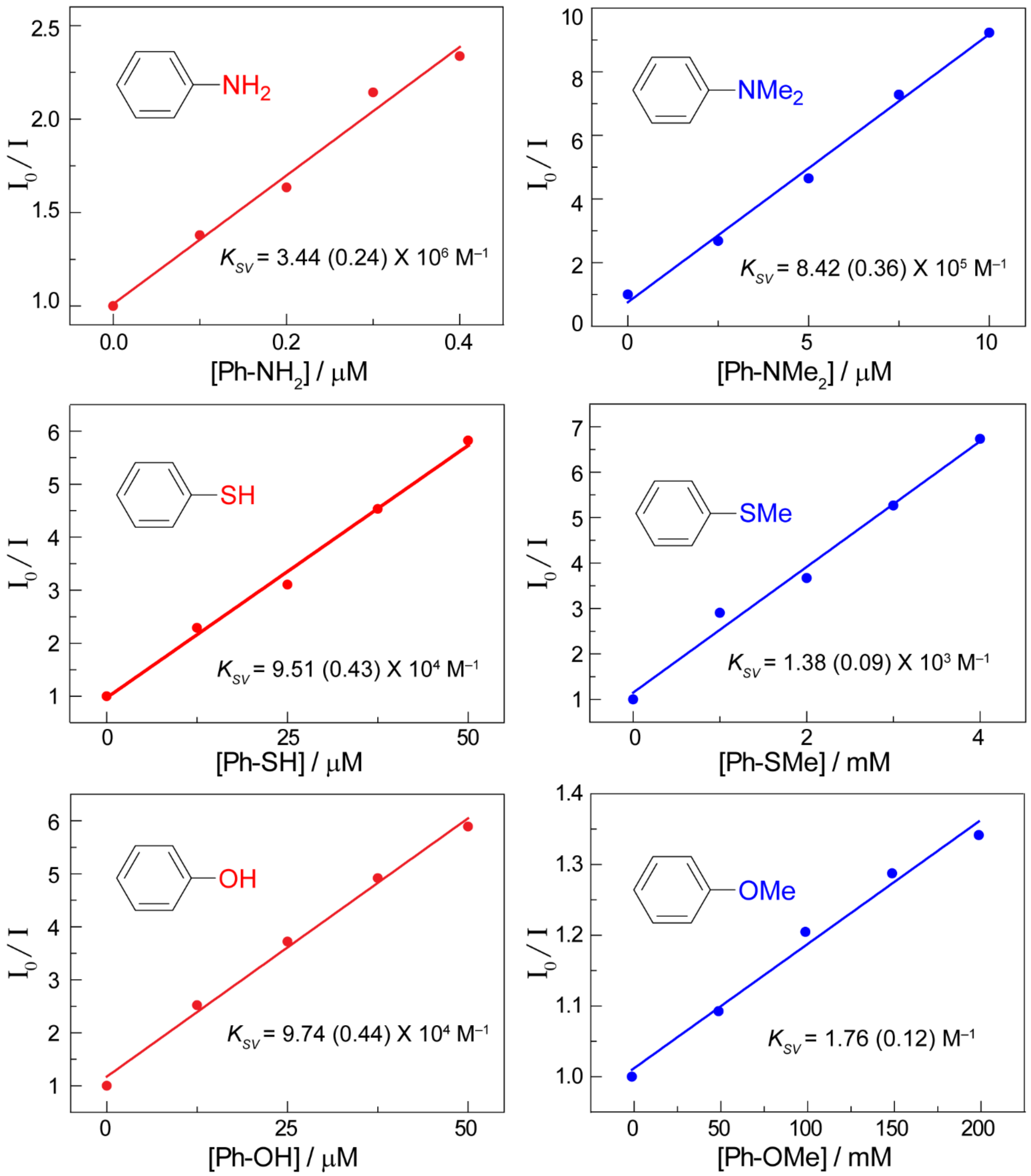

Figure S12. Stern-Volmer plots for different quenchers reacting with 1 mM **CQ** in DCM. λ_{exc} = 450 nm.

G. Photochemical CQ- and Ketone-Mediated Intramolecular Hydroamidation

A mixture of **CQ** (100 µL of a stock solution of 0.100 g **CQ** in 3 mL CD_2Cl_2, 0.02 mmol, 20 mol%), disulfide (0.01 mmol, 10 mol%), 1,4-bis(trifluoromethyl)benzene or 1,3,5-tris(trifluoromethyl)benzene as an internal standard, and amide substrate (0.10 mmol) was diluted with 0.88 mL CD_2Cl_2 to give a final concentration of 100 mM substrate. The reaction solution was transferred to a J-Young NMR tube, which was taken to the spectrometer to establish the starting ratio of substrate to internal standard. The reaction was then irradiated using a Kessil A160WE Tuna Blue LED lamp under fan cooling. After 24 h, the reaction yield was determined by ^1H NMR spectroscopy.

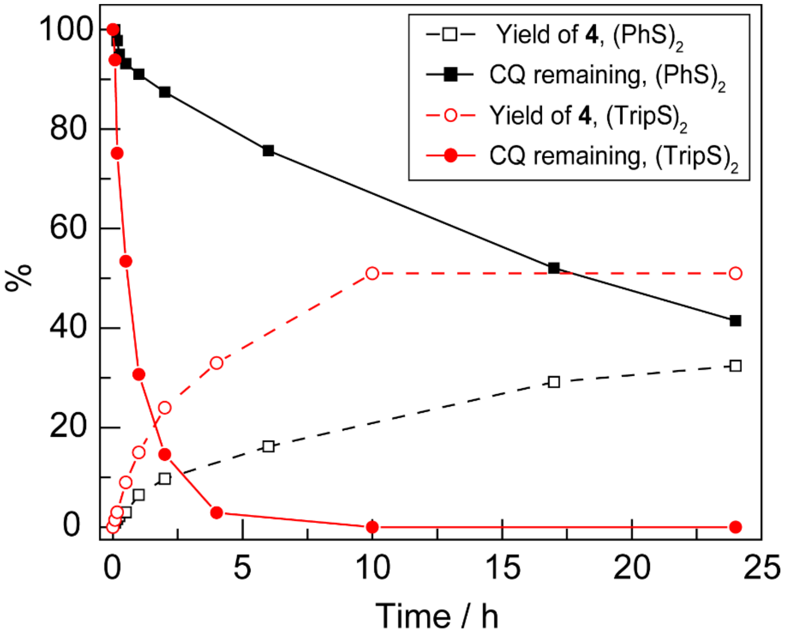

Figure S13. Time traces for the cycloamidation reaction. Time traces for the yield of cyclized product **4** (dashed lines) and % remaining of **CQ** (solid lines). Black traces are for the reaction performed with PhSSPh and red traces are with $(TripS)_2$.

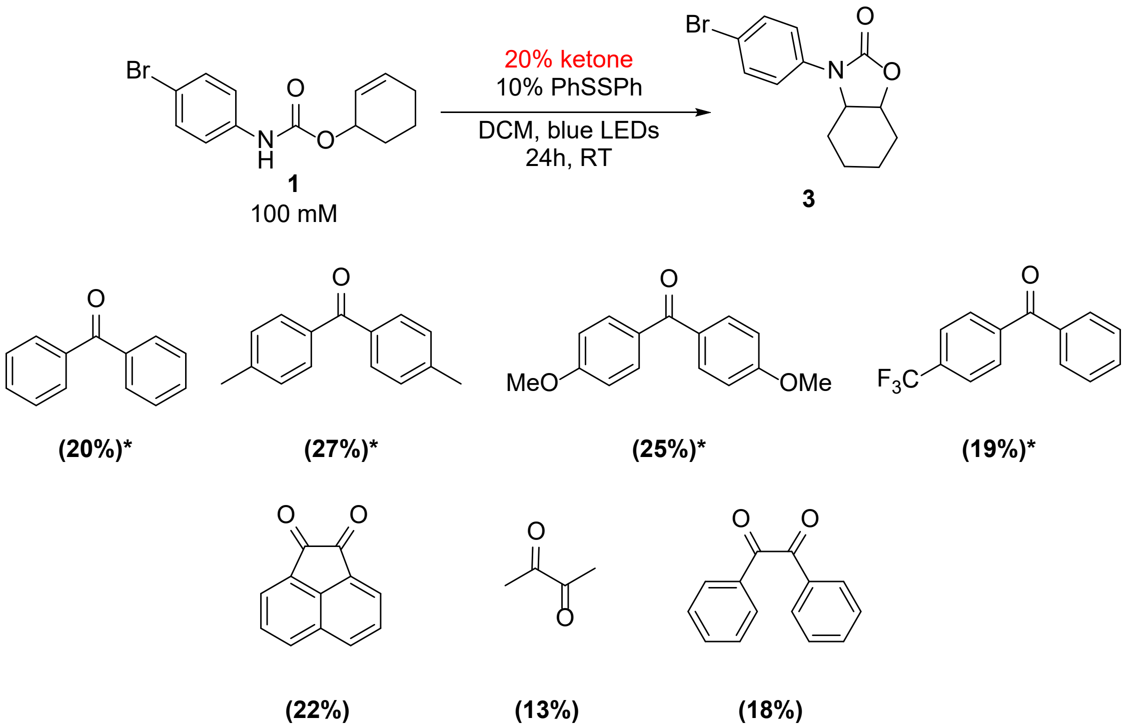

Figure S14. Photoredox intramolecular cycloamidation using various ketones as the photocatalyst. Yields as determined by ^{1}H NMR spectroscopy are denoted in parentheses. *For ketones that absorb poorly in the visible region, a 370 nm LED light source (Kessil) was used in place of the standard blue LEDs.

H. Quantum Yield Measurements

Determination of the photon flux at 467 nm. A 0.15 M solution of ferrioxalate was prepared by dissolving potassium ferrioxalate hydrate (2.210 g) in H_2SO_4 (30 mL of a 0.05 M solution). A buffered solution of 1,10-phenanthroline was prepared by dissolving 1,10-phenanthroline (0.050 g) and sodium acetate (11.25 g) in H_2SO_4 (50.0 mL of a 0.5 M solution). Both solutions were stored in the dark. To determine the photon flux of the LED (Kessil PR160-467nm), the ferrioxalate solution (3.0 mL) was placed in a cuvette and irradiated for 20 seconds at λ_{max} = 467 nm. After irradiation, the phenanthroline solution (0.53 mL) was added to the cuvette and the mixture was allowed to stir in the dark for 1 h to allow for complete coordination of ferrous ions to the phenanthroline. The absorbance of the solution was measured at 510 nm. A non-irradiated sample was also similarly prepared, and its absorbance measured at 510 nm. The difference in absorbance between the irradiated solution and the dark solution (ΔA) was calculated and used to determine the yield of Fe^{2+} according to:

$$mol\ Fe^{2+} = \frac{V \times \Delta A\ (510\ nm)}{l \times \varepsilon} = \frac{(0.00353\ L) \times (1.812 - 0.245)}{(1.00\ cm) \times (11100\ L\ mol^{-1} cm^{-1})} = 4.983 \times 10^{-7}\ mol \qquad (1)$$

where V is the total volume (0.00353 L) of the solution after addition of phenanthroline, ΔA is the difference in absorbance at 510 nm between the irradiated and non-irradiated solutions containing

added 1,10-phenantroline, l is the path length (1.00 cm), and ε is the molar absorptivity of the ferrioxalate actinometer at 510 nm (11100 L mol^{-1} cm^{-1}).

The fraction of light absorbed (f) at 467 nm by pure ferrioxalate actinometer was calculated using equation (2):

$$f = 1 - 10^{-A(467\,nm)} = 1 - 10^{-0.546} = 0.716 \tag{2}$$

Then, the photon flux was calculated using (3):

$$Photon\ flux = \frac{mol\ Fe^{2+}}{\Phi \times t \times f} = \frac{4.983 \times 10^{-7}\ mol}{0.918 \times 20.0\,s \times 0.716} = 3.791 \times 10^{-8}\ s^{-1} \tag{3}$$

where Φ is the quantum yield of ferrioxalate actinometer at 467 nm and t is the time the actinometer was irradiated.

Quantum yield measurement for hydroamidation of 1 with camphorquinone. A reaction mixture of **1** (0.148 g, 0.500 mmol), camphorquinone (17.2 mg, 0.103 mmol, 20 mol%), diphenyl disulfide (13.2 mg, 0.0604 mmol, ca. 10 mol%) and 1,4-bis(trifluoromethyl)benzene as an internal standard was dissolved in CD_2Cl_2 (5 mL). An aliquot (1 mL) was transferred to a J-Young NMR tube, which was taken to the spectrometer to establish the starting ratio of substrate to internal standard. The remaining solution (4 mL) was transferred to a cuvette containing a stir bar, which was caped, sealed with electrical tape and brought outside the glovebox to a darkroom. The reaction was then irradiated using a Kessil PR160-467nm LED lamp for 30 min. The reaction yield was determined by ^{1}H NMR spectroscopy against internal standard.
The reaction quantum yield was measured using equation (4):

$$\Phi = \frac{mol\ product}{flux \times t \times f'} = \frac{0.000006\ mol}{3.791x10^{-8}\ einstein\ s^{-1} \times 1800\,s \times 0.859} = 0.102 \tag{4}$$

Where t is the reaction time and f' is the fraction of light absorbed by camphorquinone at 467 nm (calculated as in equation 2; $A_{467nm} = 0.85$).

I. References

1. A. B. Pangborn, M. A. Giardello, R. H. Grubbs, R. K. Rosen and F. J. Timmers, *Organometallics*, 1996, **15**, 1518-1520.
2. D. C. Miller, G. J. Choi, H. S. Orbe and R. R. Knowles, *J. Am. Chem. Soc.*, 2015, **137**, 13492-13495.
3. P. G. Holder, A. A. Pizano, B. L. Anderson, J. Stubbe and D. G. Nocera, *J. Am. Chem. Soc.*, 2012, **134**, 1172-1180.
4. S. Ruccolo, Y. Qin, C. Schnedermann and D. G. Nocera, *J. Am. Chem. Soc.*, 2018, **140**, 14926-14937.
5. L. Fielding, *Tetrahedron*, 2000, **56**, 6151-6170.
6. S. G. Lias, J. E. Bartmess, J. F. Liebman, J. L. Holmes, R. D. Levin and W. G. Mallard, eds. P. J. Linstrom and W. G. Mallard, National Institute of Standards and Technology, Gaithersburg MD, 20899, ch. Ion Energetics Data.
7. Y.-R. Luo, *Handbook of Bond Dissociation Energies in Organic Compounds*, CRC press, 2002.
8. F. G. Bordwell, *Acc. Chem. Res.*, 1988, **21**, 456-463.
